# Control intervention design for preclinical and clinical trials: Consensus-based core recommendations from the third Stroke Recovery and Rehabilitation Roundtable

**DOI:** 10.1177/17474930231199336

**Published:** 2023-10-12

**Authors:** Kathryn S Hayward, Emily J Dalton, Jessica Barth, Marian Brady, Leora R Cherney, Leonid Churilov, Andrew N Clarkson, Jesse Dawson, Sean P Dukelow, Peter Feys, Maree Hackett, Steve R Zeiler, Catherine E Lang

**Affiliations:** 1 The University of Melbourne, Melbourne, VIC, Australia; 2 Providence VA Medical Center, Providence, RI, USA; 3 Glasgow Caledonian University, Glasgow, UK; 4 Shirley Ryan AbilityLab, Chicago, IL, USA; 5 University of Otago, Dunedin, New Zealand; 6 University of Glasgow, Glasgow, UK; 7 University of Calgary, Calgary, AB, Canada; 8 Reval University of Hasselt, Hasselt, Belgium; 9 University of New South Wales, Sydney, NSW, Australia; 10 Johns Hopkins University, Baltimore, MD, USA; 11 Washington University School of Medicine, St. Louis, MO, USA

**Keywords:** Clinical trial, control, preclinical, stroke, rehabilitation, recovery, consensus

## Abstract

Control comparator selection is a critical trial design issue. Preclinical and clinical investigators who are doing trials of stroke recovery and rehabilitation interventions must carefully consider the appropriateness and relevance of their chosen control comparator as the benefit of an experimental intervention is established relative to a comparator. Establishing a strong rationale for a selected comparator improves the integrity of the trial and validity of its findings. This Stroke Recovery and Rehabilitation Roundtable (SRRR) taskforce used a graph theory voting system to rank the importance and ease of addressing challenges during control comparator design. “Identifying appropriate type of control” was ranked easy to address and very important, “variability in usual care” was ranked hard to address and of low importance, and “understanding the content of the control and how it differs from the experimental intervention” was ranked very important but not easy to address. The CONtrol DeSIGN (CONSIGN) decision support tool was developed to address the identified challenges and enhance comparator selection, description, and reporting. CONSIGN is a web-based tool inclusive of seven steps that guide the user through control comparator design. The tool was refined through multiple rounds of pilot testing that included more than 130 people working in neurorehabilitation research. Four hypothetical exemplar trials, which span preclinical, mood, aphasia, and motor recovery, demonstrate how the tool can be applied in practice. Six consensus recommendations are defined that span research domains, professional disciplines, and international borders.

## Introduction

Appropriate control comparator design is critical for rigorously testing a trial hypothesis. The benefit of an experimental intervention is established relative to a pre-specified comparator. The ability to demonstrate a difference between an experimental and comparator intervention can be reduced by poor comparator choice. For example, the control comparator may include active ingredient(s) resulting in near identical interventions that will bias to the null hypothesis, or the control comparator may be unacceptable to participants resulting in a higher proportion of dropouts.^1,2^ Poor comparator design can negatively impact internal (i.e. can the outcome be causally attributed to the intervention?) and external (i.e. are the findings generalizable?) validity and the clinical utility of trial findings. Past reviews have demonstrated that little to no rationale is provided for comparator choice, and fewer words and references are used to describe a comparator as compared to the experimental intervention,^2,3^ which was reflected in scores obtained using the Template for Intervention Description and Replication (TIDieR).^4^ Consequently, the rationale for and details of the comparator control intervention in trials are often unknown. This reduces our ability to advance upon and replicate results.

Stroke recovery and rehabilitation interventions can be complex and involve multiple interacting components or ingredients.^5,6^ Careful consideration of active and inactive ingredients within a trial is required.^7,8^ How to relate controls between preclinical and/or clinical trials is also a major barrier to translation.^9^ Even a usual care comparator can pose considerable challenges, with vast variation at the micro (i.e. within and between trial sites) and macro (i.e. between countries) level.^10^ While the challenge of appropriate control comparator design has been discussed in neurorehabilitation literature,^11,12^ there is no tool available to guide decision-making. This may explain why, for example, most upper limb rehabilitation trials almost always adopt a comparator that is usual care or dose-matched usual care^2,13^ rather than a control that seeks to have all but the active ingredients of the experimental intervention present. Such a one-size-fits-all approach overlooks other comparator options that may suit the research question, trial phase,^14^ stage of stroke recovery,^15^ experimental intervention (considering active/inactive ingredients), geography (e.g. health care system and its funding), and feasibility (e.g. funding, regulatory) considerations, as well as the perspectives of people with lived experience. Establishing guidance to support appropriate selection, description, and reporting of control comparator interventions is needed.

The control taskforce of the third Stroke Recovery and Rehabilitation Roundtable (SRRRIII) aimed to understand the challenges for control comparator design; produce a tool to guide control comparator selection, description, and reporting; and provide recommendations, which if adopted, would advance the science of preclinical and clinical trials in stroke recovery and rehabilitation. Definitions used throughout are provided in Box 1. We present four hypothetical exemplars (Supplemental 1) that apply the developed CONtrol comparator deSIGN (CONSIGN) decision support tool to diverse stroke domains, intervention approaches, trial phases, recovery stages, settings, and populations. Our broad and multidisciplinary focus highlights how control comparator considerations affect all domains of stroke recovery and rehabilitation, and the broader research community involved in the conduct of preclinical and clinical trials.

**Box 1.** Definitions.

**Trial:** any research (preclinical or clinical) that prospectively assigns participants or groups of participants to one or more health-related interventions to evaluate the effects on health outcomes. This extends the National Institutes of Health (NIH) definition^16^ to include preclinical research. **Participant:** animal or human taking part in a trial. **Experimental intervention:** an intervention that manipulates the participants’ biology, behavior, and/or environment (cause) for the purpose of modifying one or more health-related biomedical or behavioral processes and/or endpoints (effect). This extends the NIH definition^16^ to encompass manipulation of not only the environment but also biology or behavior. **Control comparator (intervention)**: comparator of interest for the experimental intervention.^16^ Numerous types of control exist (Table 1). May take the form of a group, condition, or objective-criterion. **Template for Intervention Description and Replication (TIDieR)**: checklist that contains the minimum recommended items for describing an intervention^4^ which extends Item 5 of the Consolidated Standards of Reporting Trials (CONSORT) statement.^17^ **Threat:** potential alternative explanation (other than experimental intervention) for the anticipated effect or outcome that may threaten the internal validity of a trial.^18,19^ **Active ingredient**: one or more component(s) of the intervention that is(are) hypothesized to have a causal effect. This extends the Rehabilitation Treatment Specification System view^5,20^ to differentiate and describe active and inactive ingredients. **Inactive ingredient**: any additional medium or component(s) that is delivered alongside the hypothesized active ingredient component(s) that may or may not causally affect the health outcome(s).

## Methods

**Stage 1:** A working group with expertise in preclinical and clinical trial design that spanned the domains of stroke recovery including motor function, cognition, language, mood, and adjuvants, as well as biostatistics and design was established. Members were identified through Scopus searches using keywords (e.g. rehabilitation, stroke, and trial) and co-chair (KSH/CEL) knowledge of the field.
**Stage 2:** The core working group (monthly meetings, n = 13 participants) and consultants (involved as required by the core group, n = 18 participants) defined the challenges and enablers to optimal control comparator design. Various methods were used including video-conference meetings and web-based surveys. The challenges identified were ranked on a scale from 1 (lowest) to 5 (highest) by core members for both *importance* to address and *ease* of addressing during design of an optimal control comparator. A graph theory–based voting system was used to develop an overall ranked list of challenges.^21^
**Stage 3:** The CONSIGN tool was developed through two rounds of testing. All testers applied the working tool to a preclinical or clinical trial in development (real or hypothetical). In Round 1, a one-on-one approach between a core member and early career consultant researcher (n = 12) was used. In Round 2, a self-guided approach by international expert consultant researchers (n = 6) was applied. Feedback was captured using a structured form with open-ended questions: what worked well, what did not work well, what could be improved? Responses were collated and reviewed by core members and guided changes to the tool.
**Stage 4:** In a hybrid (in-person and virtual) meeting in Vienna, Austria (December 2022), we systematically addressed the challenges identified by working through the CONSIGN tool design, developing exemplar preclinical and clinical trials to demonstrate the utility of the tool and establishing recommendations for the field. Inputs from other SRRRIII taskforce group members (n > 40), as well as via a seminar plenary session at the World Congress of NeuroRehabilitation (2022, >100 attendees) were captured and used to refine ideas and the language used to express those ideas.
**Stage 5:** The CONSIGN tool was built in REDCap^22^ as a survey instrument to enable users to complete the tool iteratively for control comparator design. The tool and associated decision logic were pilot tested by four core working group members.
**Stage 6:** The core group developed four preclinical and clinical trial exemplars using the tool to support future tool users (Supplemental 1). Minor refinements were made to the tool because of the varied exemplar trials designed.
**Stage 7:** The CONSIGN tool was piloted at a workshop (n > 120 attendees from a variety of neurorehabilitation domains and clinical training background, American Society for NeuroRehabilitation Annual Meeting, Charleston, USA, April 2023) using a semi-supervised approach to complete piloting and user testing of the final version of the tool. There were 84 attempted REDCap CONSIGN tool entries. Feedback provided by attendees was used to further refine the tool for a broad audience.

## Results

**Figure 1.**
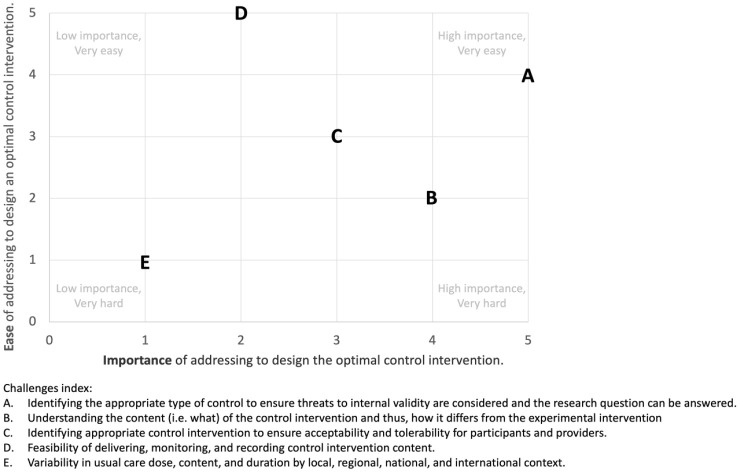
Scatter plot of aggregated rankings of identified control comparator challenges. 0 = low importance/very hard. 5 = low importance/very easy.

The aggregated ranking of five identified challenges to control comparator design, according to ease (i.e. easy to hard) and importance (i.e. low to high), are presented in Figure 1. The challenge of “identifying appropriate type of control” was ranked easy to address and very important. This confirmed the need to develop a decision support tool. Interestingly, the challenge of “variability in usual care” was ranked hard to address and of low importance. This likely reflects that it is near impossible to address usual care globally from a clinical (e.g. different health care systems and funding structures worldwide) and preclinical (e.g. different veterinary guidelines and institutional husbandry support) perspective. “Understanding the content of the control and how it differs from the experimental intervention” was ranked as very important but not easy to address despite the availability of reporting checklists, for example, TIDieR. As “feasibility of delivering, monitoring, and recording control intervention content” was ranked easy to address, it provided a possible solution to include in the trial protocol that would ensure that variability in usual care is captured.

### CONSIGN tool

The CONSIGN tool was designed and implemented to provide decision support for design and development of a control comparator for a preclinical or clinical trial. We were inclusive of both participant groups to address the standard of control design across the discovery pipeline.^14^ The tool is accessible at https://www.redcap.link/SRRR-CONSIGN and the International Stroke Recovery and Rehabilitation Alliance website https://www.strokerecoveryalliance.com/. Box 2 provides an overview of the tool. The tool addresses challenges identified including appropriate type of control selection (Step 3), threats to internal validity (Step 4), understanding the content of the control and how it differs from the experimental intervention (Step 2&6), and feasibility of the control intervention (Step 5). The tool also draws upon the established TIDieR checklist.^4^ In developing the CONSIGN tool, the core group was driven to make the content and concepts accessible to all people involved in designing a trial that explicitly compares an experimental intervention(s) to a control comparator. While conceived for stroke recovery and rehabilitation trials, we intentionally developed the tool in a way that is agnostic to domain (e.g. aphasia and motor) or population (e.g. ischaemic or haemorrhagic stroke), increasing the potential for broad utility in trial design. In doing so, people who are experienced in trials (preclinical/clinical), as well as those learning to run trials may benefit equally from using this tool.

**Box 2.** CONSIGN tool overview.

**Are you planning a preclinical or clinical trial?** *Yes*, ***continue*** *to CONSIGN tool Step 1.*	*No*, ***stop*** *using CONSIGN tool.*
**Step 1: Trial information.** • Define the research question, primary and additional hypotheses if relevant, and trial purpose and phase.	
**Step 2: Describe your experimental intervention.** • Complete the TIDieR for the trial experimental intervention, including active and inactive ingredients.	
**Step 3: Consideration of control comparator options.** • Review the listed control comparator options. • Choose all control comparator options that may address the research question. • From options, select the preferred control comparator selection. **↻ Check** if the preferred control comparator requires modification of the research question or hypothesis and amend as needed.	
**Step 4: Threats to the internal validity of your control comparator.** • Consider if common threats arising from poor control comparator selection apply to the trial and consider how each threat might impact the preferred control comparator and how each threat may be mitigated. • Identify additional threats relevant to the control comparator selection and consider how each threat might impact the preferred control comparator and how each threat may be mitigated. **↻ Check** if any threats or their mitigation requires modification of the trial research question, hypothesis, or control comparator selection, and amend as needed.	
**Step 5: Feasibility considerations.** • Identify any feasibility considerations for your preferred control comparator and how they might be mitigated. **↻ Check** if any feasibility amendments require modification of the trial research question, hypothesis, or control comparator selection, and amend as needed.	
**Step 6: Control / comparison reporting.** • Complete the TIDieR for your control comparator(s), including active and inactive ingredients. This is presented in parallel to the experimental control completed in Step 2.	
**Step 7: CONSIGN tool completion.** • On completion, download the entire CONSIGN tool as a PDF. **↻ Return to tool to review and refine** as trial planning discussions continue.	

The following sections discuss the rationale and methodological considerations for key components of the CONSIGN tool. Supplemental 2 outlines key references with an associated summary of how the reference is relevant to control comparator design.

The starting point for control comparator selection is the research question (Step 1). The most appropriate comparator is one that allows a researcher to answer their specific research question. For example, if the research question asks if combination treatment “A + B” is better than “A” or “B” alone, then the comparator to “A + B” needs to be “A” alone and “B” alone. As another example, if the research question asks if the combination “A + B” is better than usual care, then the comparator ought to be usual care (Table 1).

**Table 1.** Types of control comparator.

Type	Definition	More useful if . . .	Less useful if . . .
For trials with a control arm:			
Placebo	A group who receives a fake substance or treatment which is designed to have no known value (e.g., pill designed to look like the experimental one without the active ingredient)	✓Evaluating pharmaceutical agents	✗ It may be impossible to conceal an active ingredient or method in a rehabilitation trial
Vehicle	A group who receives only the substance (e.g., saline, gel) that is used to deliver the experimental compound	✓Evaluating pharmaceutical agents ✓Preclinical studies	✗ It may be impossible to conceal an active ingredient or method in a rehabilitation trial ✗ Not evaluating a topical or injectable agent
Sham	A group who receives a pretend or fake procedure	✓Evaluating interventions that include surgical or other procedures e.g., non-invasive brain stimulation devices	✗ Not evaluating specific procedures ✗ The sham poses unacceptable risk
No training/intervention	A group that intentionally receives no training or no intervention	✓No training or intervention is the standard of care in the study environment ✓Aim of study is to compare the intervention to nothing	✗ May be unethical for many conditions where there is at least some rehabilitation care typically provided
Basic, standard conventional, prescribed, traditional, usual, or usual and customary care	A collection of labels that imply other, generic care in the control group. Care may be from a variety of disciplines. The labels generally describe what services (e.g., medical, rehabilitation, nursing, social services) a person might receive if they were not in the trial. This could range from no care to care that overlaps with the experimental intervention, depending on a multitude of factors. Variations in what is delivered could be individual, local, regional, or national	✓Primary aim is to determine if the experimental intervention is “better than usual care”	✗ Preclinical studies ✗ Usual care in the study environment is unknown or varies widely across study sites ✗ Usual care includes part or all of the active ingredients being evaluated in the experimental intervention
Protocolised	An additional label indicating that health care providers must follow a specific protocol	✓Long duration and/or multisite studies to minimize heterogeneity across sites, therapists and investigators and the potential for overlap with the experimental active ingredients, dosing, and/or delivery mode	✗ If no aspect of the experimental intervention is ever Protocolised in clinical practice
Guideline-based	A group that receives best-evidence care as described in clinical practice guidelines	✓Aim is to compare the experimental intervention to best known care.	✗ If there is no guideline available
Dose-matched or equivalent	A group that receives the same intervention dose of an alternative or different treatment. Dose could be matched on various dimensions^23^ e.g., repetitions, time in therapy	✓Aim is to evaluate the experimental intervention, and rule out confounding effects of dose	✗ Aim is to specifically evaluate dose of an intervention
Wait-list or delayed treatment	A group that waits to receive the experimental intervention until a later time	✓To preserve study retention, since all participants eventually get the experimental intervention ✓An aim is to evaluate a critical period for delivery of the experimental intervention	✗ The study environment might change over time, potentially confounding the ability to deliver the same intervention (e.g., global pandemic) ✗ The condition being studied fluctuates over time ✗ For some interventions (e.g., psychological) it can discourage people from seeking usual care
Attention	A group that receives generic contact, controlling for the number and duration of interactions with the trial staff or healthcare providers	✓When there is a possibility that outcomes may change simply based on time and interaction with study personnel	✗ When it is not possible to provide a therapeutic rationale for the nature of the attention control
Active	Often used as an additional label to describe a group that receives a comparable, standard intervention that contains different active ingredients	✓Primary study aim evaluates the benefit of one intervention vs. another	✗ The two chosen interventions evaluated share substantial overlap of active ingredients
Historical	A group derived from an available data set of animals or humans who did not receive the experimental intervention	✓Often for early phase trials	✗ Primary study aim is efficacy ✗ Health care environment from the available data set has changed with time or is different from that where the intervention will take place
For trials with a control condition:			
Objective criterion	An outcome value derived from available dataset of animals or humans who did not receive the experimental intervention	✓For early phase single arm trials only	✗ Primary study aim is efficacy ✗ Health care environment from the available data set has changed with time or is different from that where the intervention will take place
Within participant	An outcome value derived as a difference between the intervention and an appropriate comparator condition for the same participant	✓Provides tighter control for confounders as individual participants serve as their own control ✓Suitable for any trial phase	✗ Health care environment or individual participants state are assumed not to change from control condition to appropriate comparator

Defining the content of the experimental intervention is key to knowing what needs to be controlled (Step 2). TIDieR^1^ is an established tool to guide the reporting of intervention content and is a recognized extension to Item 5 of CONSORT.^9^ We made one modification to TIDieR by expanding the “what” section. Our tool specifically requires users to define known or hypothesized active and inactive ingredients of the experimental intervention (Box 1). This modification addresses the established notion that it is commonplace in pharmacological trials to define active and inactive ingredients, but it is not standard in non-pharmacological trials, for example, a behavioral intervention.^7^

Selecting the type of control is critical to enable researchers to demonstrate that the outcome observed is due to the experimental intervention as opposed to many other, alternative explanations (Step 3). This can be achieved by ensuring that the comparator intervention is as similar as possible to the experimental intervention in all aspects except the active ingredient(s). There are other considerations that may come into play during selection, such as the ability to conceal active ingredients, availability of evidence of effective treatments or clinical practice guidelines, acceptability of the control comparator for people with lived experience, as well as regulatory and ethical requirements. Table 1 provides an overview of possible control comparator options and contextualizes the considerations for when each may (or may not) be useful. While the list provides distinct options, they can and often are used in combination, for example, *placebo vehicle, protocolised usual care*, or *active attention control.*

**Figure 2.**
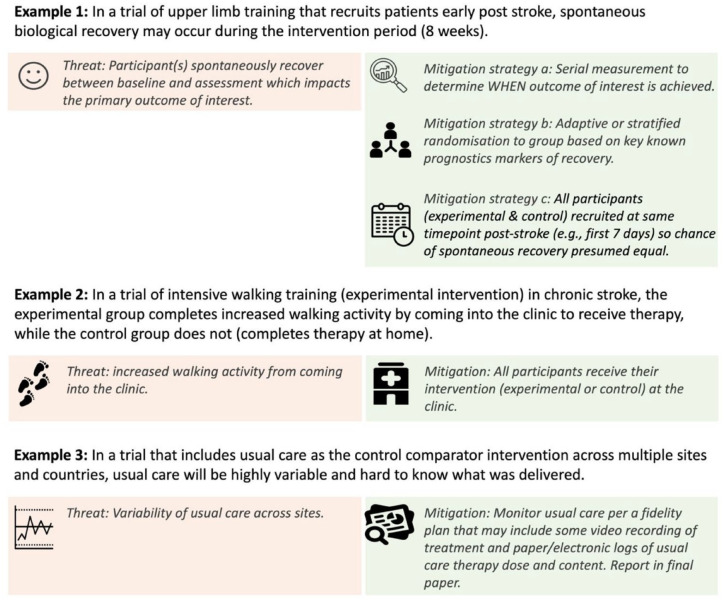
Demonstration of how threats may impact control comparator selection and potential mitigation strategies.

Careful consideration of alternative explanations for the outcome of interest other than the experimental intervention must be considered during control comparator design (Step 4). Such alternative explanations can threaten trial internal validity; hence they are termed threats^18,19^ (Box 1). Alternate explanations may occur before the trial begins or during the conduct of the trial, for example, because of differences in the natural trajectory of recovery (e.g. the rate of spontaneous recovery may be different in the experimental and control groups due to timing of enrollment); participants exhibiting a learning effect as they become familiar with the outcome measure (e.g. a cognitive outcome was performed on more of the experimental group just before the study started as compared to the control); how the intervention is delivered (e.g. at the clinic for experimental while at home for the control); or the rate of dropout (e.g. the control intervention is considered a waste of time and many participants dropout). Considering all possible alternative explanations a priori means the control comparator can be designed to minimize potential threats using appropriate mitigation strategies. Figure 2 provides an overview of some common threats and options for mitigation.

The CONSIGN tool was deliberately structured to design for rigor first and adjust for feasibility (Step 5) later, after selecting the preferred control comparator type that considers all possible threats. Addressing feasibility later in the tool enables the trial design team to understand the implications of altering their control comparator design due to such considerations. Feasibility considerations may relate to access to participants, funding, regulatory policies, and trial sponsors.

In the final step, the tool directs users to complete a control comparator TIDieR (Step 6). For this step, the completed experimental TIDieR (Step 3) is visually presented alongside the control TIDieR for comparison. Inclusion of an independent TIDieR for the control comparator(s) specifically addresses a major gap in current practice^3^ and provides the trial design team with materials that can be used as supplemental data in any trial publication.

To demonstrate CONSIGN tool utility, we present four exemplar trials in Supplemental 1 and provide an overview of each in Table 2. The variability in exemplars demonstrates application to different trial phases,^14^ domains,^24^ phases of recovery,^15^ control comparator considerations (Table 1), threats to internal validity (Figure 2), and feasibility constraints.

**Table 2.** Overview of the four exemplar that apply the CONSIGN tool to control comparator design.

	Exemplar 1	Exemplar 2	Exemplar 3	Exemplar 4
Research question/aim	Does Cogenamp^a^ and rehabilitation training improve cognitive outcomes assessed by standard means compared to either Cogenamp alone, rehabilitative training alone or no training?	To determine whether cognitive behavioral therapy (CBT) prevents depression after stroke in community dwelling individuals better than an attention control (non-therapeutic).	Does U-CAN^b^ result in better outcomes (improved naming in conversation) than no treatment or computer treatment with non-linguistic stimuli?	Does the combination of Recoverall^c^ and Gaitercise^d^ improve gait post-stroke more than Recoverall or Gaitercise or Usual Care?
Trial phase	Preclinical	Phase II	Phase II	Phase III
Domain of interest	Cognition	Mood	Language	Motor
Time post stroke^e^	Early subacute	Early and late subacute	Chronic	Acute
Experimental	Cogenamp^a^	Cognitive behavioral therapy	U-CAN^b^	Recoverall^c^ + Gaitercise^d^
Control comparator	Sham stroke (and no training); Stroke alone (no training); Stroke + Protocolized training alone; Stroke + vehicle to deliver placebo; Stroke + vehicle to deliver Cogenamp; Stroke + Protocolized training + vehicle to deliver placebo.	Attention control (non-therapeutic)	No treatment, wait-list or placebo computer treatment with non-linguistic stimuli	Recoverall or Gaitercise or Usual Care

CONSIGN: CONtrol DeSIGN.

a Cogenamp is a hypothetical, novel, insulin-like growth factor 1 agent.

b U-CAN is a hypothetical, novel computer naming treatment for aphasia.

c Recoverall is a hypothetical, novel drug to enhance central nervous system plasticity.

d Gaitercise is a hypothetical protocolised gait and exercise training program.

e Time post stroke per Stroke Recovery and Rehabilitation Roundtable definitions.^15^

### Recommendations

Box 3 outlines recommendations that address the identified challenges (Figure 1), support the use of the CONSIGN tool for control comparator design, promote control comparator monitoring, and uptake of the TIDieR^4^ reporting guideline.

**Box 3.** Control comparator design recommendations.

Use the SRRR CONSIGN tool in trial design to support control comparator development
Collaborate with biostatisticians, clinical, and methodological experts, as well as people with lived experience to optimize control comparator design.
Select an optimal control comparator that specifically addresses the research question of interest and statement of hypothesis, controls for the active ingredients of the experimental intervention, and mitigates possible threats to internal validity.
Describe the planned control comparator and the process for monitoring intervention adherence and fidelity in the trial protocol using TIDieR or an equivalent standard.
Describe the actual control comparator and the extent to which it was delivered as planned in a published trial report using TIDieR or an equivalent standard.
Document and report information specific to local, regional, and/or national systems of care to contextualize the control comparator, aid generalization to other settings, and facilitate comparison to existing literature.

## Discussion

Rigorously testing an experimental intervention and the associated primary hypothesis requires an appropriate control comparator design. The CONSIGN tool was developed to address the identified challenges (Figure 1) and provides decision support for design of a control comparator for preclinical and clinical trials. This tool steps the user through important considerations during the design process. It does not provide a single “perfect” control comparator design on completion because of the heterogenous nature of research questions and the potential for feasibility considerations. From the outset, we agreed to align preclinical and clinical control comparator design to avoid repeating past mistakes, as well as aid translation of intervention testing from preclinical to clinical populations.^25^ Although developed for stroke recovery and rehabilitation trials, the utility of the tool extends to other trial populations and interventions, since the structure of the tool is not condition or content specific. It is important to note that CONSIGN complements existing guidelines for reporting standards for comparative trials including CONSORT^17^ and TIDieR^t4^, as well as previous tools produced by the SRRRs including the Trial Development Framework that guides trialists to establish GO, NO-GO decision points important to overall trial design.^26^

A key lesson learned during this consensus process was that there is no “one-size-fits-all” control comparator selection that can be applied to all stroke recovery and rehabilitation trials. Stroke recovery and rehabilitation interventions are often complex, and active ingredients may be hard to identify. We acknowledge that this makes designing a control comparator more difficult than selecting a comparator in a pharmacological trial. Many stroke recovery and rehabilitation trials have adopted “usual care” or “dose-matched usual care” as the control comparator.^2,13^ It is possible that this approach has not yielded breakthrough intervention(s) because the (a) active ingredients of the usual care comparator intervention(s) overlap with the experimental, and/or (b) heterogeneity in usual care produces heterogeneity in outcomes that in turn limit the ability to detect between-groups differences. There may also be funding or regulatory considerations that have driven the control comparator selection used in prior trials. While in many fields, it may be feasible to test an experimental intervention against a placebo (e.g. a pharmacological trial), where there is no active substance or treatment provided, this is often impractical in rehabilitation research, and may be unethical as it might require modification or withdrawal of standard care. Indeed, usual stroke care can have a large impact on functional outcomes.^3^ In recognizing this, CONSIGN as a tool provides a breadth of available control comparator types for consideration (Table 1), which can be used individually or collectively. Extending beyond the initial trial design stage, we urge researchers, funding agencies, and publishers to carefully consider control comparator design during manuscript and grant review processes. We encourage researchers to include the CONSIGN tool in supplemental materials to demonstrate why a particular control comparator was selected.

The application of the CONSIGN tool to four exemplars demonstrates the broad utility of this tool. The challenge of appropriate control comparator design exists across domains, including interventions that target cognition (Exemplar 1), mood (Exemplar 2), language (Exemplar 3), and motor (Exemplar 4) outcomes. We recognize there may be different considerations when selecting a type of control depending on the domain, for example, a waitlist control may be inappropriate as it may discourage people with mood disorders from seeking help, or that guideline care may not be possible for new and emerging scientific areas or geographical regions that have yet to establish clinical practice guidelines. Distinct opportunities may be available to preclinical trials, such as using a higher number of control comparators to systematically evaluate an intervention. To increase translatability, preclinical controls should explicitly report control conditions and attempt to mimic clinical controls. For example, preclinical investigations often allow larger differences between experimental and control and may even allow non-treated control arms.^9^ To overcome this gap, preclinical trials should use some form of clinically inspired best practice.^25^

The use of the CONSIGN tool does not negate the need for input of trial methodologists, biostatisticians, clinicians, and people with lived experience on the research team. As design of the experimental intervention evolves during trial preparation, it is highly likely that the needs of the control comparator design may also change. We view control comparator design as an iterative process where drafts of the CONSIGN tool output can be brought back to the research team (including people with lived experience) repeatedly until there is agreement on a rigorous and feasible control comparator condition(s) that appropriately addresses the research question of interest and statement of hypothesis, and controls for the active ingredients of the experimental intervention and possible threats to internal validity.

Improved planning and reporting of control comparators, including usual care, will ultimately facilitate the ability to synthesize findings across trials. The current heterogeneity of stroke trial control comparator types and lack of information about their content means that data syntheses often group different interventions with different active ingredients together. Sharing the content of the experimental and control comparator interventions using the TIDieR,^4^ or an equivalent standard, will greatly enhance future research synthesis efforts.

## Conclusion

This SRRR used expert consensus to identify the current challenges with control comparator design, and developed recommendations that span research domains, professional disciplines, and international borders to move the field forward. An important product emerging from this effort is the CONSIGN tool to guide control comparator selection, description, and reporting. This tool is designed to stimulate critical thinking about control comparator design but does not provide a single “perfect” control comparator design on completion. Adhering to the recommendations can improve control comparator design and ultimately establish a stronger rationale for a selected comparator. This has the potential to foster translation of trial outcomes that deliver benefits for people living with stroke. Our group looks forward to continuing to develop and improve the utility of the tool.

## Supplemental Material

sj-docx-1-wso-10.1177_17474930231199336 – Supplemental material for Control intervention design for preclinical and clinical trials: Consensus-based core recommendations from the third Stroke Recovery and Rehabilitation Roundtable Click here for additional data file. Supplemental material, sj-docx-1-wso-10.1177_17474930231199336 for Control intervention design for preclinical and clinical trials: Consensus-based core recommendations from the third Stroke Recovery and Rehabilitation Roundtable by Kathryn S Hayward, Emily J Dalton, Jessica Barth, Marian Brady, Leora R Cherney, Leonid Churilov, Andrew N Clarkson, Jesse Dawson, Sean P Dukelow, Peter Feys, Maree Hackett, Steve R Zeiler and Catherine E Lang in International Journal of Stroke

sj-pdf-2-wso-10.1177_17474930231199336 – Supplemental material for Control intervention design for preclinical and clinical trials: Consensus-based core recommendations from the third Stroke Recovery and Rehabilitation Roundtable Click here for additional data file. Supplemental material, sj-pdf-2-wso-10.1177_17474930231199336 for Control intervention design for preclinical and clinical trials: Consensus-based core recommendations from the third Stroke Recovery and Rehabilitation Roundtable by Kathryn S Hayward, Emily J Dalton, Jessica Barth, Marian Brady, Leora R Cherney, Leonid Churilov, Andrew N Clarkson, Jesse Dawson, Sean P Dukelow, Peter Feys, Maree Hackett, Steve R Zeiler and Catherine E Lang in International Journal of Stroke
