# Supplementary material for: Control intervention design for preclinical and clinical trials: Consensus-based core recommendations from the third Stroke Recovery and Rehabilitation Roundtable
Source: Int J Stroke. 2023 Oct 12;19(2):169–79. doi: 10.1177/17474930231199336 (PMC10811967; doi:10.1177/17474930231199336)
Supplement: sj-docx-1-wso-10.1177_17474930231199336 – Supplemental material for Control intervention design for preclinical and clinical trials: Consensus-based core recommendations from the third Stroke Recovery and Rehabilitation Roundtable [file sj-docx-1-wso-10.1177_17474930231199336.docx]

**SUPPLEMENTAL MATERIAL**

**Title:** Control intervention design for preclinical and clinical trials: Consensus-based core recommendations from the third Stroke Recovery and Rehabilitation Roundtable

**Table of contents:**

[**Supplemental 1:** SRRR CONtrol comparator DeSIGN [CONSIGN] Tool applied to hypothetical trial exemplars 2](#_Toc134274721)

[Exemplar 1 Preclinical cognitive trial 3](#_Toc134274722)

[Exemplar 2 Clinical mood trial 18](#_Toc134274723)

[Exemplar 3 Clinical language trial 30](#_Toc134274724)

[Exemplar 4 Clinical pharmacological and exercise trial 43](#_Toc134274725)

[**Supplemental 2:** Key references with statement related to control comparator design. 57](#_Toc134274726)

[**Supplemental 3:** Disclosures unrelated to the published materials**.** 59](#_Toc134274727)

**https://redcap.link/SRRR-CONSIGN**

**Corresponding Author:** Kathryn S Hayward, PhD, [0000-0001-5240-3264](about:blank). Departments of Physiotherapy, Medicine (RMH), and Florey Institute of Neuroscience and Mental Health, University of Melbourne, Melbourne, Victoria, Australia. 161 Barry St, Carlton Victoria Australia, 3053. Phone +61 03 9035 7293. Email: [kate.hayward@unimelb.edu.au](mailto:kate.hayward@unimelb.edu.au)

# Supplemental 1: SRRR CONtrol comparator DeSIGN [CONSIGN] Tool applied to hypothetical trial exemplars

**Note these exemplars are hypothetical trials (and interventions).*

## Exemplar 1 Preclinical cognitive trial

Preclinical hypothetical pharmacological study to improve cognition outcomes in the acute recovery epoch post-stroke.

Rationale: Insulin-like Growth Factor I (IGF-I) has been shown to play an important part in normal brain development, promoting neuronal growth, regulating plasticity amongst other mechanisms. However, as we age, IGF-1 levels decrease and low IGF-I levels have previously been shown to correlate with poor recovery after stroke. Whilst rehabilitation is known to induce a trophic factor response, including increasing IGF-1 levels, a threshold response required to achieve desired physiological responses are often not reached. Therefore, the overarching aim here is to test the therapeutic efficacy of the newly synthesized IGF-1 mimetic in concert with rehabilitation to further enhance post-stroke cognitive recovery. Note, this trial uses Cogenamp as a hypothetical IFG-1 mimetic.

*Please note that the output below may appear slightly different to the PDF you can download from REDCap. The questions are consistent, but small changes have been made to formatting for publication purposes.*

| Are you undertaking a trial? | ⭘ No  **⮾ Yes, a preclinical trial**  ⭘ Yes, a clinical trial |
| --- | --- |

| **Step 1: Research question(s) and hypotheses**  *The research question is a critical motivator of control intervention design. In this section, you will explicitly specify the research question (or research aim, depending on how you prefer to phrase it) and hypotheses you intend to evaluate. At the end of Step 1, you will decide to proceed or exit the tool.* |
| --- |

| What is your **research question**(s)? | Does the combination of Cogenamp and rehabilitation training improve cognitive outcomes more than rehabilitation training alone in a mouse model of stroke to the pre-frontal cortex? |
| --- | --- |
| What is your **primary hypothesis**? | Cogenamp and rehabilitation training will improve cognitive outcomes assessed by standard means compared to either Cogenamp alone, rehabilitative training alone or no training. |
| Do you have additional hypotheses? | ⭘ No  **⮾ Yes** |
|  | Combined treatment (Cogenamp and rehabilitation) will have a greater effect in females compared to male mice. |

| **You may not need this tool if:**  *Your study is PURELY focused on feasibility. This means your question/aim is to examine the ability to conduct your study (e.g. recruitment, ability to deliver the intervention, retention, etc.) and NOT to demonstrate a causal link between the intervention and the outcome. If this is true, you may not need this tool as it predominantly addresses the selection of a control condition for causal studies. You plan to use an Objective Performance Criteria (OPC). An OPC is a numerical target value derived from historical data and may be used in a dichotomous (pass/fail) manner for review and comparison of safety, or effectiveness endpoints. If this is true, you may not need this tool as your comparison will be made against an OPC rather that a control condition. To learn more: Objective performance criteria, Section 7.6.1.*  **You need this tool if:**  *You plan to* ***explicitly evaluate a causal link between the experimental intervention and the outcome of interest****. This means you are trying to test if the intervention leads to the outcome of interest. This includes the examination of safety, dose, efficacy, effectiveness, or a combination thereof. This may also include an examination of the feasibility of the control intervention(s). If this is true, continue completing this tool because you will need to select a control condition.* |
| --- |

| Do you wish to continue with this tool? | ⭘ No  **⮾ Yes** |
| --- | --- |

**Step 2: Experimental Intervention Description**

*There are elements of your experimental intervention that can impact the selection of your control intervention. The TIDieR is an established tool to guide the reporting of intervention content. In this step, you will work through the elements of your experimental intervention using a modified version of the TIDieR.*

| **Corresponding TIDieR Item** | **Question** | **Experimental Intervention Description** |
| --- | --- | --- |
| TIDieR – Item 1  [Brief Name] | What is the **name or phrase** that labels your experimental intervention? | (1) Cogenamp + (2) Rehabilitation Training |
| TIDieR – Expansion item^  [WHAT] | What are the known or hypothesized **active ingredients** of your experimental Intervention? | (1) Cogenamp; proprietary molecule acting at the site of the stroke.  (2) Rehabilitation Training; administration of forelimb (e.g., reaching tasks) and hindlimb exercises (e.g., running wheel, ladder) to promote repetitive rehabilitation practice. |
| TIDieR – Expansion item^  [WHAT] | What are the known or hypothesized **inactive ingredients** of your experimental intervention? | (1) Cogenamp - Hydrogel; a porous, permeable solid formed in situ once injected into the stroke core. The hydrogel allows for the slowed delivery of the active ingredient (Cogenamp) from the stroke core into the per-infarct region.  (2) Rehabilitation training - food-driven rewards to participate in rehabilitation training. |
| TIDieR – Item 2  [WHY] | What is the **rationale** behind your active and/or inactive ingredients? | (1) Cogenamp - Proprietary molecule designed to enhance post-stroke cognition. Hydrogel - Designed to encapsulate Cogenamp and slowly release it from within the stroke core.  (2) Rehabilitation training - Signal of benefit from both previous preclinical and clinical studies that rehabilitation training, in particular exercise training, can improve cognition after stroke. |
| TIDieR – Item 3  [WHAT] | What **materials** will be part of your experimental interventions? | All mice: Housing cages. Feeding wells. Food. Water. Mice vivarium that meets lighting and temperature requirements consistent with protocol.  (1) Cogenamp – The required amount of drug to administer. Apparatus to inject.  (2) Rehabilitation training; Running wheel, Ladder, Plexiglas reaching apparatus, palatable mini M&Ms. |
| TIDieR – Item 4  [WHAT] | What **procedures** will be part of your experimental interventions? | (1) Cogenamp – Surgery will be performed aseptically. Focal ischemia will be created by injecting endothelin-1. Endothelin infusion will run for 2 minutes and the needle will remain in place for 3 minutes following the infusion to minimize backflow. Under aseptic surgical procedures, hydrogel containing either Cogenamp or the vehicle used to make up Cogenamp, will be injected into the stroke cavity 5 days post-stroke. The Hydrogel infusion will run for ~10 minutes and the needle will remain in place for 2 minutes following infusion to minimize backflow. Body temperature will be maintained at ~37 degrees during all procedures.  (2) Rehabilitation training; Trial staff will move mice into their own individual training care (vivarium) for rehabilitation training (see below for further details). Once rehabilitation training is completed, trial staff will move mice back to standard housing in groups of 2 or 3. |
| TIDieR – Item 5  [WHO PROVIDED] | **Who** will provide the experimental intervention? | Trained trial staff will provide all intervention aspects including the Cogenamp and Rehabilitation Training. These individuals will be blinded to the condition. They will have > 5 years of experience delivering pharmacological and training interventions to mice and have completed a 60-minute training session on the protocol that covers both how to inject hydrogel and administer the rehabilitation training. |
| TIDieR – Item 6  [HOW] | **How will** the experimental intervention be provided? | (1) Cogenamp - The trained trial staff will inject the hydrogel containing either placebo (saline) or Cogenamp.  (2) The trained trial staff will administer the rehabilitation training as per the protocol. They will move the mice into the training cage (vivarium) and there will be one mouse in each training cage. |
| TIDieR – Item 7  [WHERE] | **Where** will the experimental intervention be provided? | Delivery of all interventions (Cogenamp and Rehabilitation Training) will occur in the mice vivarium under low light condition to minimize stress, and under standardized temperature and humidity. No animals shall leave the vivarium. |
| TIDieR – Item 8  [WHEN and HOW MUCH] | **When and how much** of the experimental intervention will be provided? | (1) Cogenamp; will be provided at 2mg at a single time point that is 7 days post-stroke.  (2) Rehabilitation Training; The intervention will be provided 1x2hr session per day, 5 days per week (2 days rest) for 4 weeks. |
| TIDieR – Item 9  [TAILORING] | **How will** the experimental intervention be tailored? | No personalization, titration or adaptions are planned for either intervention (Copenamp or rehabilitation training). |
| TIDieR – Item 11  [HOW WELL - PLANNED] | **How well will** the experimental intervention be provided? | (1) Cogenamp; Injection of hydrogel can be assessed using endogenous fluorescent signal. Tissue penetration and residual hydrogel concentrations can be assessed using HPLC.  (2) Rehabilitation training; all training sessions will be recorded and reviewed for fidelity. |
| **Note that TIDieR Item 10 [MODIFICATIONS] and TIDieR Item 12 [HOW WELL – ACTUAL] are not included as trial intervention is yet to be administered.*  *^As described in the main paper, expansion of the WHAT section to include active and inactive ingredients.* | | |

| **Step 3. Consideration of comparator condition options**  *Now that you have information about your trial question(s), hypotheses, and experimental intervention, we will probe deeper into potential comparator conditions.*  *As you are explicitly comparing a control condition, you need to select the type of control that might be best. As you hope to demonstrate that the health outcome is due to the experimental intervention (as opposed to many other, alternative explanations), you must explicitly exclude other alternative explanations.*  *This can be achieved by making sure that the comparator condition is as similar as possible to the experimental condition in all aspects except the active ingredients.*  *In this step, you will consider various comparator options and if or how they might be suitable for your trial.* |
| --- |

| Is your comparator: | **⮾ at the group level (most common choice)**  ⭘ at the individual level (e.g., matching)  ⭘ within participant* (the participant is their own control)  **The “within participant” option is suitable when there is no underlying trend over time* |
| --- | --- |

| *Now that you have information about your trial question(s), hypotheses, and experimental intervention, we will probe deeper into potential comparator conditions.*  *You may already be considering several comparator conditions. The tool outlines a range of comparator conditions available, using 11+ different labels. A definition of each type of comparator, along with when it may or may not be useful is provided to help you narrow your selection.*  *After going through this information, you may select multiple options for what you are considering. Note that some options are typically used as additional descriptors of other options. For example, protocolized + usual care, would indicate a comparator group that gets usual care that follows a specific, agreed upon trial protocol.*  *See Table 2 in the main paper for complete information.* |
| --- |

| Which comparators do you want to consider: | **⮾ Placebo**  **⮾ Vehicle**  **⮾ Sham**  **⮾ No training / No intervention**  ⭘ Usual care  **⮾ Protocolized**  ⭘ Guideline based  ⭘ Dose-matched / dose equivalent  ⭘ Waitlist / delayed treatment  ⭘ Attention only  ⭘ Active  ⭘ Historical |
| --- | --- |
| What is your working preferred control comparator(s)? | **Group 1,** Control Comparator: Sham stroke *(sham and no training/intervention)*  **Group 2,** Control Comparator: Stroke alone *(no training/intervention).*  **Group 3,** Control Comparator: Stroke + Protocolized rehabilitation training alone.  **Group 4,** Control Comparator: Stroke + Hydrogel (vehicle) to deliver saline (placebo).  **Group 5,** Control Comparator: Stroke + Hydrogel (vehicle) to deliver Cogenamp.  **Group 6,** Control Comparator: Stroke + Protocolized rehabilitation training + hydrogel (vehicle) to deliver saline (placebo).  **Note,* ***Group 7*** *is the experimental intervention which will receive Stroke + Protocolized rehabilitation training + hydrogel (vehicle) to deliver Cogenamp.* |
| Does the comparator(s) you selected require you to modify your research question or hypotheses? | ⭘ Yes  **⮾ No** |

*Explanation: It is important to note that preclinical studies permit this very thorough and comprehensive causal investigation that cannot be done in clinical populations. This affords the inclusion of multiple control comparator groups.*

| **Step 4. Threats to internal validity**  *Now that you have information about your trial question(s), hypotheses, and experimental intervention, we will probe deeper into potential comparator conditions.*  *As you are explicitly comparing an experimental intervention to a control condition in order to evaluate a causal link, you need to carefully anticipate possible, alternative explanations for the health outcome, other than the experimental intervention. Alternative explanations are important as they can threaten the internal validity of your trial. That is why they are termed threats. Alternate explanations may occur before the trial begins or during the conduct of the trial. Considering all possible alternative explanations now means the comparator for the trial can be designed to minimize potential threats using various strategies.*  *Designing an appropriate control group mitigates some of the threats to internal validity (so called single group threats) but some other threats (so called multiple group threats) may remain even in presence of an appropriate design. Therefore, in this step, you will consider how various common threats to stroke recovery and rehabilitation trials may or may not impact your control group. Here, the intentional focus is on threats that could arise from inadequate comparator selection. We have not included every possible threat to the trial itself. This will help you develop a working list for further discussion with your trial team, including a statistician.*  *See Figure 1 in the main paper for more information.* |
| --- |

| Is **spontaneous recovery** a threat for your trial? | **⮾ Yes**  ⭘ No |
| --- | --- |
| *What strategies will you use to mitigate spontaneous recovery during the intervention?* | Mice recruited at the same time point post-stroke. |
| Is an **unacceptable or uncredible comparator selection** a threat to your trial? | ⭘ Yes  **⮾ No** |
| *What strategies will you use to mitigate an unacceptable or uncredible compactor intervention?* | Not applicable |
| Is **increased withdrawal from the control group** because of randomization a threat for your trial? | ⭘ Yes  **⮾ No** |
| *What strategies will you use to mitigate participant withdrawal from the control group?* | Not applicable |
| Is not knowing or **not controlling the content of the comparator** group a threat to your trial? | ⭘ Yes  **⮾ No** |
| *What strategies will you use to mitigate the unknown or uncontrolled comparator intervention?* | Not applicable |
| Does the **act of coming into a clinic** a threat for your trial? | ⭘ Yes  **⮾ No** |
| *What strategies will you use to mitigate increased participant activity due to simply coming into the clinic?* | Not applicable |
| What **additional threat(s)** do you have and what strategies will you use to mitigate those threats? | (1) Rehabilitative training could account for the majority or all the cognitive protection. Mitigation: Have a control group that receives only rehabilitative interventions and no hydrogel and/or drug.  (2) Hydrogel injection could account for the majority or all the cognitive protection. Mitigation: Have a control group that receives only hydrogel injection and no rehabilitative training and/or drug.  (3) The time of day that the rehabilitative intervention is provided could influence outcomes. Mitigation: Rehabilitative interventions begin during the transition from day into night for all animals.  (4) Day-to-day variability such that different days might influence outcomes despite no change in the experimental conditions (e.g. noise outside the lab, atmospheric pressure, or other nuanced changes in environment sensed by mice). Mitigation: Use of a Randomized Block (RB) design. Testing will be done at the same time of day and in the same testing rooms that have been designed to minimize the influence of external factors (noise, temperature etc) |

| **You have identified the following threats and strategies to internal validity:**   - Spontaneous recovery: Mice recruited at the same time point post-stroke. - Not credible or acceptable comparator: Not appliable - Increased comparator group withdrawal: Not appliable - Unknown or uncontrolled comparator intervention: Not appliable - Increased activity from coming into the clinic*:* Not applicable. - Additional threats and strategies: - (1) Rehabilitative training could account for the majority or all of the cognitive protection. Mitigation: Have a control group that receives only rehabilitative interventions and no hydrogel and/or drug. - (2) Hydrogel injection could account for the majority or all of the cognitive protection. Mitigation: Have a control group that receives only hydrogel injection and no rehabilitative training and/or drug. - (3) The time of day that the rehabilitative intervention is provided could influence outcomes. Mitigation: Rehabilitative interventions begin during the transition from day into night for all animals. - (4) Day-to-day variability, such that different days might influence outcomes despite no change in the experimental conditions (e.g. noise outside the lab, atmospheric pressure, and/or other nuanced changes in the environment sensed by mice). Mitigation: Use of a Randomized Block (RB) design. Testing will be undertaken at the same time of day and in the same testing rooms that have been designed to minimise the influence of external factors (noise, temperature etc) |
| --- |

| Do any of these **threats** require you to **modify your research question or hypotheses?** | ⭘ Yes  **⮾ No** |
| --- | --- |
| Do any of these **threats** require you to **modify your comparator condition selection(s)?** | ⭘ Yes  **⮾ No** |
| Your preferred control: | **Group 1,** Control Comparator: No stroke *(sham and no training/intervention)*  **Group 2,** Control Comparator: Stroke alone *(no training/intervention).*  **Group 3,** Control Comparator: Stroke + Protocolized rehabilitation training alone.  **Group 4,** Control Comparator: Stroke + Hydrogel (vehicle) to deliver saline (placebo).  **Group 5,** Control Comparator: Stroke + Hydrogel (vehicle) to deliver Cogenamp.  **Group 6,** Control Comparator: Stroke + Protocolized rehabilitation training + hydrogel (vehicle) to deliver saline (placebo). |

| **Step 5: Feasibility Considerations**  *You are now ready to ponder if the design and comparator conditions you have selected will be feasible to execute in your trial. In this step, you will be asked how various potential feasibility considerations may prompt you to reconsider your selections.* |
| --- |

| Do you have feasibility considerations related to **access to participants**? | ⭘ Yes  **⮾ No** |
| --- | --- |
| *How does considerations due to access to participants impact your prior selections in this tool?* | Not applicable |
| Do you have feasibility considerations related to **funding**? | **⮾ Yes**  ⭘ No |
| *How does considerations due to funding impact your prior selections in this tool?* | Cost of conducting the trial given the number of control comparator groups. |
| Do you have feasibility considerations related to the **trial sponsor**? | ⭘ Yes  **⮾ No** |
| *How does considerations due to funding impact your prior selections in this tool?* | Not applicable |
| Do you have feasibility considerations related to **regulatory policies**? | ⭘ Yes  **⮾ No** |
| *How does considerations due to regulatory policies impact your prior selections in this tool?* | Not applicable |
| Do you have any **additional feasibility** considerations? | Not applicable |

| **You have identified the following feasibility concerns:**   - Access issues: Not applicable - Funding issues: Cost of conducting the trial given the number of control comparator groups. - Sponsor issues: Not applicable - Regulatory issues: Not applicable - Other feasibility issues: Not applicable |
| --- |

| Do any of your feasibility considerations require you to **modify your comparator** condition selection(s)? | ⭘ Yes  **⮾ No** |
| --- | --- |

| **Step 6: Comparator TIDieR(s)**  *You now have information about your research question, hypothesis, experimental intervention, comparator condition(s), threats and strategies to mitigate identified threats, and any adjustments for feasibility.*  *In this step, you will be asked to complete the TIDieR for your comparator condition(s). Many studies have a single comparator condition, but some research questions and hypothesis will require multiple comparator conditions. Preclinical studies often have multiple comparators to probe the biological mechanisms along with causality.  This tool allows for up to 6 comparator conditions.  If you are doing a human clinical trial and you have more than 3 comparator conditions, go back and reconsider your research question and hypotheses, in consultation with your trial design expert.* |
| --- |

| **How many** comparator conditions will you have? | ⭘ 1  ⭘ 2  ⭘ 3 | ⭘ 4  ⭘ 5  **⮾ 6** | ⭘ 7  ⭘ 8  ⭘ 9 | ⭘ 10 |
| --- | --- | --- | --- | --- |

| **Corresponding TIDieR Item** | **Question** | **Intervention Description** | | | | | | |
| --- | --- | --- | --- | --- | --- | --- | --- | --- |
|  |  | **Experimental** *(from Step 2)* | **Comparator 1** | **Comparator 2** | **Comparator 3** | **Comparator 4** | **Comparator 5** | **Comparator 6** |
| TIDieR – Item 1  [Brief Name] | What is the **name or phrase** that labels your intervention? | (1) Cogenamp +  (2) Rehabilitation Training | Sham stroke  **Sham and no training / intervention* | Stroke  **No training / intervention* | Stroke + Rehabilitation Training *(protocolized)*  **No drug intervention* | Stroke + Hydrogel *(vehicle)* to deliver saline *(placebo)*  **No training* | Stroke + Hydrogel *(vehicle)* to deliver Cogenamp.  **No training* | Stroke +  Rehabilitation training *(protocolized)* + Hydrogel *(vehicle)* to  deliver saline *(placebo)*. |
| TIDieR – Expansion item^  [WHAT] | What are the known or hypothesized **active ingredients** of your Intervention? | (1) Cogenamp; proprietary molecule acting at the site of the stroke.  (2) Rehabilitation Training (protocolized); administration of forelimb (e.g., reaching tasks) and hindlimb exercises (e.g., running wheel, ladder) to promote repetitive rehabilitation practice. | No active ingredients as no training/ intervention will be provided. | No active ingredients as no training/ intervention will be provided. | Rehabilitation Training – See experimental intervention TIDieR. | Hydrogel - See experimental intervention TIDieR.  Placebo - No active ingredients. | Hydrogel - See experimental intervention TIDieR.  Cogenamp - See experimental TIDieR. | Rehabilitation Training – See experimental intervention TIDieR.  Hydrogel - See experimental intervention TIDieR.  Placebo - No active ingredients. |
| TIDieR – Expansion item^  [WHAT] | What are the known or hypothesized **inactive ingredients** of your intervention? | (1) Cogenamp - Hydrogel; a porous, permeable solid formed in situ once injected into the stroke core. The hydrogel allows for the slowed delivery of the active ingredient (Cogenamp) from the stroke core into the per-infarct region.  (2) Rehabilitation training - food-driven rewards to participate in rehabilitation training. | Incidental exercise around training cage. | Incidental exercise around training cage. | Rehabilitation Training – See experimental intervention TIDieR. | Hydrogel - See experimental intervention TIDieR.  Placebo - No inactive ingredients. | Cogenamp - Hydrogel; a porous, permeable solid formed in situ once injected into the stroke core. The hydrogel allows for the slowed delivery of the active ingredient (Cogenamp) from the stroke core into the per-infarct region. | Rehabilitation training - food-driven rewards to participate in rehabilitation training. |
| TIDieR – Item 2  [WHY] | What is the **rationale** behind your active and/or inactive ingredients? | (1) Cogenamp - Proprietary molecule designed to enhance post-stroke cognition. Hydrogel - Designed to encapsulate Cogenamp and slowly release it from within the stroke core.  (2) Rehabilitation training - Signal of benefit from both previous preclinical and clinical studies that rehabilitation training, in particular exercise training, can improve cognition after stroke. | Mice will not receive any training /intervention - therefore no active ingredients are hypothesized or known. | Mice will not receive any training /intervention - therefore no active ingredients are hypothesized or known. | Rehabilitation Training – See experimental intervention TIDieR. | Hydrogel - See experimental intervention TIDieR. | Cogenamp - See experimental intervention TIDieR. | Rehabilitation training - See experimental intervention TIDieR. |
| TIDieR – Item 3  [WHAT] | What **materials** will be part of your intervention? | All mice: Housing cages. Feeding wells. Food. Water. Mice vivarium that meets lighting and temperature requirements consistent with protocol.  (1) Cogenamp – The required amount of drug to administer. Apparatus to inject.  (2) Rehabilitation training; Running wheel, Ladder, Plexiglas reaching apparatus, palatable and mini M&Ms. | See experimental intervention TIDieR for materials for all mice. | See experimental intervention TIDieR for materials for all mice. | See experimental intervention TIDieR for materials for all mice.  Rehabilitation Training – See experimental intervention TIDieR. | See experimental intervention TIDieR for materials for all mice.  Hydrogel - Apparatus to inject.  Placebo – the required amount of saline to administer. | See experimental intervention TIDieR for materials for all mice.  Cogenamp – see experimental intervention TIDieR | See experimental intervention TIDieR for materials for all mice.  Rehabilitation training – see experimental intervention TIDieR  Hydrogel - Apparatus to inject.  Placebo – the required amount of saline to administer. |
| TIDieR – Item 4  [WHAT] | What **procedures** will be part of your intervention? | (1) Cogenamp – Surgery will be performed aseptically. Focal ischemia will be created by injecting endothelin-1. Endothelin infusion will run for 2 minutes and the needle will remain in place for 3 minutes following the infusion to minimize backflow. Under aseptic surgical procedures, hydrogel containing either Cogenamp or the vehicle used to make up Cogenamp, will be injected into the stroke cavity 5 days post-stroke. The Hydrogel infusion will run for ~10 minutes and the needle will remain in place for 2 minutes following infusion to minimize backflow. Body temperature will be maintained at ~37 degrees during all procedures.  (2) Rehabilitation training; Trial staff will move mice into their own individual training care (vivarium) for rehabilitation training (see below for further details). Once rehabilitation training is completed, trial staff will move mice back to standard housing in groups of 2 or 3. | Sham - Surgery will be performed aseptically. Needle will be inserted into hemisphere and remain in place for a consistent amount of time to experimental procedure but no injection to create focal ischemia will occur. Body temperature will be maintained at ~37 degrees.  No training – mice will remain in standard housing in groups of 2 or 3. | Stroke - Surgery will be performed aseptically. Focal ischemia will be created by injecting endothelin-1. Endothelin infusion will run for 2 minutes and the needle will remain in place for 3 minutes following infusion to minimize backflow. Body temperature will be maintained at ~37 degrees.  No training – see comparator 1 TIDieR | Stroke – see Comparator 2 description  Rehabilitation training – see experimental TIDieR. | Stroke – see Comparator 2 description  Hydrogel and saline - Hydrogel containing saline will be injected into the stroke cavity 5-days post-stroke under aseptic conditions. Hydrogel infusion will run for ~10 minutes and the needle will remain in place for 2 minutes following infusion to minimize backflow. Body temperature will be maintained at ~37 degrees.  No training – see comparator 1 TIDieR | See Experimental Intervention (Cogenamp) TIDieR  No training – see comparator 1 TIDieR | Stroke – see Comparator 2 description  Experimental Intervention (hydrogel + saline) TIDieR  Rehabilitation training – see experimental TIDieR. |
| TIDieR – Item 5  [WHO PROVIDED] | **Who** will provide the intervention? | Trained trial staff will provide all intervention aspects including the Cogenamp and Rehabilitation Training. These individuals will be blinded to the condition. They will have > 5 years of experience delivering pharmacological and training interventions to mice and have completed a 60-minute training session on the protocol that covers both how to inject hydrogel and administer the rehabilitation training. | Mice will not receive any intervention training but will be cared for by trained lab staff. | Mice will not receive any intervention training but will be cared for by trained lab staff. | See experimental intervention TIDieR. | See experimental intervention TIDieR | See experimental intervention TIDieR | See experimental intervention TIDieR |
| TIDieR – Item 6  [HOW] | **How will** the intervention be provided? | (1) Cogenamp - The trained trial staff will inject the hydrogel containing either placebo (saline) or Cogenamp.  (2) The trained trial staff will administer the rehabilitation training as per the protocol. They will move the mice into the training cage (vivarium) and there will be one mice in each training cage. | Mice will not receive any intervention training | Mice will not receive any intervention training | See experimental intervention TIDieR. | See experimental intervention TIDieR. | See experimental intervention TIDieR | See experimental intervention TIDieR |
| TIDieR – Item 7  [WHERE] | **Where** will the intervention be provided? | Delivery of all interventions (Cogenamp and Rehabilitation Training) will occur in the mice vivarium under low light condition to minimize stress, and under standardized temperature and humidity. No animals shall leave the vivarium. | Mice will not receive any intervention training | Mice will not receive any intervention training | See experimental intervention TIDieR | See experimental intervention TIDieR | See experimental intervention TIDieR | See experimental intervention TIDieR |
| TIDieR – Item 8  [WHEN and HOW MUCH] | **When and how much** of the intervention will be provided? | (1) Cogenamp; will be provided at 2mg at a single time point that is 7 days post-stroke.  (2) Rehabilitation Training; The intervention will be provided 1x2hr session per day, 5 days per week (2 days rest) for 4 weeks. | Mice will not receive any intervention training | Mice will not receive any intervention training | See experimental intervention TIDieR. | Placebo - (saline) will be provided at same does as the Cogenamp (2mg dose, once, 7 days post-stroke) using the Hydrogel as consistent vehicle. | Cogenamp - See experimental intervention TIDieR | Rehabilitation training – see experimental intervention TIDieR  Placebo - (saline) will be provided at same does as the Cogenamp (2mg dose, once, 7 days post-stroke) using the Hydrogel as consistent vehicle. |
| TIDieR – Item 9  [TAILORING] | **How will** the intervention be tailored? | No personalisation, titration or adaptions are planned for either intervention (Copenamp or rehabilitation training). | Mice will not receive any intervention training. | Mice will not receive any intervention training. | No personalisation, titration or adaptions are planned for this control comparator. | No personalisation, titration or adaptions are planned for this control comparator. | No personalisation, titration or adaptions are planned for this control comparator. | No personalisation, titration or adaptions are planned for this control comparator. |
| TIDieR – Item 11  [HOW WELL - PLANNED] | **How well will** the intervention be provided? | (1) Copenamp; Injection of hydrogel can be assessed endogenous fluorescent signal. Tissue penetration and residual hydrogel concentrations can be assessed using HPLC.  (2) Rehabilitation training; all training sessions will be recorded and reviewed for fidelity. | Mice will not receive any intervention training | Mice will not receive any intervention training | See experimental intervention TIDieR. | Hydrogel - See experimental intervention TIDieR  Placebo – will be monitor as per Copenamp in the experimental intervention TIDieR. | Copenamp - See experimental intervention TIDieR | Rehabilitation training - See experimental intervention TIDieR.  Hydrogel - See experimental intervention TIDieR.  Placebo – will be monitor as per Copenamp in the experimental intervention TIDieR. |
| **Note that TIDieR Item 10 [MODIFICATIONS] and TIDieR Item 12 [HOW WELL – ACTUAL] are not included as trial intervention is yet to be administered.*  *^As described in the main paper, expansion of the WHAT section to include active and inactive ingredients.* | | | | | | | | |

| **Congratulations, you have completed all the items in the tool!**  *If you choose the "Save & Return Later" button, you will be given a randomly generated access code to re-enter the tool and revise your answers.*  *If you chose the "Submit" button, you will be given the option to download a PDF document with your answers AND a randomly-generate access code to re-enter the tool and revise your answers.* |
| --- |

**SRRR CONtrol comparator DeSIGN [CONSIGN] Tool**

## Exemplar 2 Clinical mood trial

Phase II clinical trial of a hypothetical behavioral (non-pharmacological) intervention to prevent post-stroke depression in the early subacute recovery epoch.

Rationale: Approximately half of people recovering from stroke will experience depression or depression symptoms of clinical concern in the first year of recovery. In the context of depression, social conversations with another person may be an active ingredient and thus poses numerous challenges for comparator selection when doing nothing is not possible. This exemplar aims to highlight how the tool might support design of an active control comparator.

*Please note that the output below may appear slightly different to the PDF you can download from REDCap. The questions are consistent, but small changes have been made to formatting for publication purposes.*

| Are you undertaking a trial? | ⭘ No  ⭘ Yes, a preclinical trial  **⮾ Yes, a clinical trial** |
| --- | --- |

| **Step 1: Research question(s) and hypotheses**  *The research question is a critical motivator of control intervention design. In this section, you will explicitly specify the research question (or research aim, depending on how you prefer to phrase it) and hypotheses you intend to evaluate. At the end of Step 1, you will decide to proceed or exit the tool.* |
| --- |

| What is your **research question**(s)? | Determine whether cognitive behavioral therapy (CBT) prevents depression after stroke in community-dwelling individuals better than an attention control (non-therapeutic)? |
| --- | --- |
| What is your **primary hypothesis**? | CBT is an effective method of preventing depression after stroke in community dwelling individuals compared to attention control (non-therapeutic). |
| Do you have additional hypotheses? | **⮾ No**  ⭘ Yes |

| **You may not need this tool if:**  *Your study is PURELY focused on feasibility. This means your question/aim is to examine the ability to conduct your study (e.g. recruitment, ability to deliver the intervention, retention, etc.) and NOT to demonstrate a causal link between the intervention and the outcome. If this is true, you may not need this tool as it predominantly addresses the selection of a control condition for causal studies. You plan to use an Objective Performance Criteria (OPC). An OPC is a numerical target value derived from historical data and may be used in a dichotomous (pass/fail) manner for review and comparison of safety, or effectiveness endpoints. If this is true, you may not need this tool as your comparison will be made against an OPC rather that a control condition. To learn more: Objective performance criteria, Section 7.6.1.*  **You need this tool if:**  *You plan to* ***explicitly evaluate a causal link between the experimental intervention and the outcome of interest****. This means you are trying to test if the intervention leads to the outcome of interest. This includes the examination of safety, dose, efficacy, effectiveness, or a combination thereof. This may also include an examination of the feasibility of the control intervention(s). If this is true, continue completing this tool because you will need to select a control condition.* |
| --- |

| Do you wish to continue with this tool? | ⭘ No  **⮾ Yes** |
| --- | --- |

**Step 2: Experimental Intervention Description**

*There are elements of your experimental intervention that can impact the selection of your control intervention. The TIDieR is an established tool to guide the reporting of intervention content. In this step, you will work through the elements of your experimental intervention using a modified version of the TIDieR.*

| **Corresponding TIDieR Item** | **Question** | **Experimental Intervention Description** |
| --- | --- | --- |
| TIDieR – Item 1  [Brief Name] | What is the **name or phrase** that labels your experimental intervention? | Cognitive Behavioural Therapy (CBT) |
| TIDieR – Expansion item^  [WHAT] | What are the known or hypothesized **active ingredients** of your experimental Intervention? | Cognitive Behavioural Therapy (CBT); Content; Structure; Delivery (including workbook); Active participation; Talking; Time spent in a social situation |
| TIDieR – Expansion item^  [WHAT] | What are the known or hypothesized **inactive ingredients** of your experimental intervention? | Travel; Using a computer/device |
| TIDieR – Item 2  [WHY] | What is the **rationale** behind your active and/or inactive ingredients? | Active: CBT is a type of talking therapy that is designed to help modify thinking patterns and change mood and behaviors. CBT is one of the most evidence-based psychological interventions for treating depression in the general population. There is limited evidence of effectiveness in people with stroke. For research purposes, it is structured over a pre-specified number of weeks, sessions of a pre-specified topic and duration and workbooks/homework for participants. CBT can be delivered via telephone, online, face-to-face; one to one or in group sessions. The duration of interaction with others might be significantly more than usual. Mixing socially with other people and the length of time spent doing this. Outcome assessments (asking about mood) may be considered an active aspect of the trial/intervention.  Inactive: Time, structure and social aspects need controlling. An exchange of utterances between two or more people without the intent of modifying thinking patterns or behaviors. |
| TIDieR – Item 3  [WHAT] | What **materials** will be part of your experimental interventions? | Workbook/homework: Can be paper-based and/or online. Computer/device: When CBT is delivered via telephone or online. Private space(s): For therapist and participant(s) regardless of whether they are conducing CBT via telephone, online or face-to-face. |
| TIDieR – Item 4  [WHAT] | What **procedures** will be part of your experimental interventions? | At time on consent, the participant selects their preferred model of intervention delivery (see below for details). The trial therapist then provides them with a timetable outlining when the sessions would occur throughout the experimental period. For those participants who select telephone/online delivery, they will be provided with a number or Zoom link to accommodate the sessions and this process will be trialed prior to the commencement of therapy. If face to face (individual or group) is selected, then participant will be shown where the treatment will occur and how they can access the building prior to experimental intervention commencing. |
| TIDieR – Item 5  [WHO PROVIDED] | **Who** will provide the experimental intervention? | Trained, supervised, culturally competent healthcare professional (nurse, psychologist, other), who completes and passes all trial-related CBT training and assessment(s), and is willing and able to be supervised.  Training: GCP, protocol/trial procedures, CBT program manual, workbook. |
| TIDieR – Item 6  [HOW] | **How will** the experimental intervention be provided? | All modes allowed: Telephone, online, face-to-face, group and individual as per the preference of the participant, as long as in a private space. |
| TIDieR – Item 7  [WHERE] | **Where** will the experimental intervention be provided? | Office space for therapist with telephone/computer for telephone/online delivery and sufficient room to conduct one to one or small-group (up to 4 people) sessions. |
| TIDieR – Item 8  [WHEN and HOW MUCH] | **When and how much** of the experimental intervention will be provided? | There will be 6 manualized CBT sessions to be completed over 12 weeks with a minimum of six days between sessions. Each session is designed to take up to 60 minutes. The shortest time the sessions may be completed is within 6 weeks, the maximum time is 12 weeks. |
| TIDieR – Item 9  [TAILORING] | **How will** the experimental intervention be tailored? | No tailoring |
| TIDieR – Item 11  [HOW WELL - PLANNED] | **How well will** the experimental intervention be provided? | Sessions will be recorded and assessed for adherence to the protocol by the delegated investigator. When therapists are found to deviate from the protocol, they will be required to attend re-training with the delegated investigator, which may be tailored specifically to the deviation rather than repeat the full training if appropriate. They will be required to pass a retraining-related assessment. |
| **Note that TIDieR Item 10 [MODIFICATIONS] and TIDieR Item 12 [HOW WELL – ACTUAL] are not included as trial intervention is yet to be administered.*  *^As described in the main paper, expansion of the WHAT section to include active and inactive ingredients.* | | |

| **Step 3. Consideration of comparator condition options**  *Now that you have information about your trial question(s), hypotheses, and experimental intervention, we will probe deeper into potential comparator conditions.*  *As you are explicitly comparing a control condition, you need to select the best control type. As you hope to demonstrate that the health outcome is due to the experimental intervention (as opposed to many other, alternative explanations), you must explicitly exclude other alternative explanations.*  *This can be achieved by ensuring that the comparator condition is as similar as possible to the experimental condition in all aspects except the active ingredients. In this step, you will consider various comparator options and if or how they might be suitable for your trial.* |
| --- |

| Is your comparator: | **⮾ at the group level (most common choice)**  ⭘ at the individual level (e.g., matching)  ⭘ within participant* (the participant is their own control)  **The “within participant” option is suitable when there is no underlying trend over time* |
| --- | --- |

| *Now that you have information about your trial question(s), hypotheses, and experimental intervention, we will probe deeper into potential comparator conditions.*  *You may already be considering several comparator conditions. The tool outlines a range of comparator conditions available, using 11+ different labels. A definition of each type of comparator, along with when it may or may not be useful is provided to help you narrow your selection.*  *After going through this information, you may select multiple options for what you are considering. Note that some options are typically used as additional descriptors of other options. For example, protocolized + usual care, would indicate a comparator group that gets usual care that follows a specific, agreed upon trial protocol.*  *See Table 2 in the main paper for complete information.* |
| --- |

*Explanation: For older people who did not have an active social life before stroke, participating in an active intervention where you regularly discuss your recovery and other topics after stroke may be a substantial change from before stroke. This is why an attention control intervention is required to ensure the 'social' aspects of CBT are controlled for.*

| Which comparators do you want to consider: | **⮾ Placebo / vehicle**  ⭘ Sham  ⭘ No training / No intervention  **⮾ Usual care**  ⭘ Protocolized  ⭘ Guideline based  ⭘ Dose-matched / dose equivalent  ⭘ Waitlist / delayed treatment  **⮾** **Attention only**  **⮾ Active**  ⭘ Historical |
| --- | --- |
| What is your working preferred control comparator(s)? | Attention control |
| Does the comparator(s) you selected require you to modify your research question or hypotheses? | ⭘ Yes  ⮾ No |

| **Step 4. Threats to internal validity**  *Now that you have information about your trial question(s), hypotheses, and experimental intervention, we will probe deeper into potential comparator conditions.*  *As you are explicitly comparing an experimental intervention to a control condition in order to evaluate a causal link, you need to carefully anticipate possible, alternative explanations for the health outcome, other than the experimental intervention. Alternative explanations are important as they can threaten the internal validity of your trial. That is why they are termed threats. Alternate explanations may occur before the trial begins or during the conduct of the trial. Considering all possible alternative explanations now means the comparator for the trial can be designed to minimize potential threats using various strategies.*  *Designing an appropriate control group mitigates some of the threats to internal validity (so called single group threats) but some other threats (so called multiple group threats) may remain even in presence of an appropriate design. Therefore, in this step, you will consider how various common threats to stroke recovery and rehabilitation trials may or may not impact your control group. Here, the intentional focus is on threats that could arise from inadequate comparator selection. We have not included every possible threat to the trial itself. This will help you develop a working list for further discussion with your trial team, including a statistician.*  *See Figure 1 in the main paper for more information.* |
| --- |

| Is **spontaneous recovery** a threat for your trial? | **⮾ Yes**  ⭘ No |
| --- | --- |
| *What strategies will you use to mitigate spontaneous recovery during the intervention?* | In this case, depression often arises or is exacerbated post-stroke, so randomization stratification factor related to time post-stroke. This mitigation strategy helps to partially address this threat, continued monitoring for depression during the trial is also warranted. |
| Is an **unacceptable or uncredible comparator selection** a threat to your trial? | ⭘ Yes  **⮾ No** |
| *What strategies will you use to mitigate an unacceptable or uncredible compactor intervention?* | Not applicable |
| Is **increased withdrawal from the control group** because of randomization a threat for your trial? | **⮾ Yes**  ⭘ No |
| *What strategies will you use to mitigate participant withdrawal from the control group?* | The attention control group will receive useful generic content to people after stroke - just no intentionally therapeutic content designed to prevent depression |
| Is not knowing or **not controlling the content of the comparator** group a threat to your trial? | ⭘ Yes  **⮾ No** |
| *What strategies will you use to mitigate the unknown or uncontrolled comparator intervention?* | Not applicable |
| Does the **act of coming into a clinic** a threat for your trial? | **⮾ Yes**  ⭘ No |
| *What strategies will you use to mitigate increased participant activity due to simply coming into the clinic?* | It is not only increased activity, but also the fact that the requirement to travel may prevent some people from participating. As a result, the intervention may be provided without clinic visits via telehealth if preferred/needed. |
| What **additional threat(s)** do you have and what strategies will you use to mitigate those threats? | - |

| **You have identified the following threats and strategies to internal validity:**   - Spontaneous recovery: In this case, depression often emerges over time post stroke, so randomization should include a stratification factor related to time post stroke. - Not credible or acceptable comparator: Not applicable - Increased comparator group withdrawal: The attention control group will receive useful, generic content to people after stroke - just no intentionally therapeutic content designed to prevent depression. - Unknown or uncontrolled comparator intervention: Not applicable. - Increased activity from coming into clinic*:* It is not only increased activity, but also the fact that the requirement to travel may prevent some people from participating. As a result, the intervention may be provided without clinic visits via telehealth if preferred/needed. - Additional threats and strategies: For older people who did not have an active social life before stroke, participating in an active intervention where you regularly discuss your recovery and other topics after stroke may be a substantial change from before stroke. This is why an attention control intervention is required to ensure the 'social' aspects of CBT are controlled for. |
| --- |

| Do any of these **threats** require you to **modify your research question or hypotheses?** | ⭘ Yes  **⮾ No** |
| --- | --- |
| Do any of these **threats** require you to **modify your comparator condition selection(s)?** | ⭘ Yes  **⮾ No** |
| Your preferred control: | **Attention control** |

| **Step 5: Feasibility Considerations**  *You are now ready to ponder if the design and comparator conditions you have selected will be feasible to execute in your trial. In this step, you will be asked how various potential feasbility considerations may prompt you to reconsider your selections.* |
| --- |

| Do you have feasibility considerations related to **access to participants**? | **⮾ Yes**  ⭘ No |
| --- | --- |
| *How does considerations due to access to participants impact your prior selections in this tool?* | Availability or access to participants – trial design allows for community-dwelling participants to self-nominate for trial consideration. Options  to participate over Zoom or phone may allow for more people to participate. |
| Do you have feasibility considerations related to **funding**? | **⮾ Yes**  ⭘ No |
| *How does considerations due to funding impact your prior selections in this tool?* | Funding for the trial is required. Will need to ensure that the funding mechanism provided sufficient funds to cover control group costs. |
| Do you have feasibility considerations related to the **trial sponsor**? | ⭘ Yes  **⮾ No** |
| *How does considerations due to funding impact your prior selections in this tool?* | Not applicable. |
| Do you have feasibility considerations related to **regulatory policies**? | ⭘ Yes  **⮾ No** |
| *How does considerations due to regulatory policies impact your prior selections in this tool?* | Not applicable. |
| Do you have any **additional feasibility** considerations? | Delivery of the CBT interventions requires specialized knowledge and skills. Other trials issues, such as: trained therapist availability and consistency, trained people to deliver attention control consistently, and non-adherence to trial protocol by therapist and attention control "people" will need to be monitored closely. |

| **You have identified the following feasibility concerns:**   - Access issues: Availability or access to participants - trial design allows for community-dwelling participants to self-nominate for trial consideration. Options to participate over Zoom or phone may allow for more people to participate. - Funding issues: Funding for the trial is required. Will need to ensure that the funding mechanism provided sufficient funds to cover costs. - Sponsor issues: Not applicable - Regulatory issues: Not applicable - Other feasibility issues: Delivery of the CBT interventions requires specialized knowledge and skills. Other trials issues, such as: trained therapist availability and consistency, trained people to deliver attention control consistently, and non-adherence to trial protocol by therapist and attention control "people" will need to be monitored closely |
| --- |

| Do any of your feasibility considerations require you to **modify your comparator** condition selection(s)? | ⭘ Yes  **⮾ No** |
| --- | --- |

| **Step 6: Comparator TIDieR(s)**  *You now have information about your research question, hypothesis, experimental intervention, comparator condition(s), threats and strategies to mitigate identified threats, and any adjustments for feasibility.*  *In this step, you will be asked to complete the TIDieR for your comparator condition(s). Many studies have a single comparator condition, but some research questions and hypothesis will require multiple comparator conditions.* *Preclinical studies often have multiple comparators to probe the biological mechanisms along with causality.  This tool allows for up to 6 comparator conditions.  If you are doing a human clinical trial and you have more than 3 comparator conditions, go back and reconsider your research question and hypotheses, in consultation with your trial design expert.* |
| --- |

| **How many** comparator conditions will you have? | **⮾ 1**  ⭘ 2  ⭘ 3 |
| --- | --- |

| **Corresponding TIDieR Item** | **Question** | **Intervention Description** | |
| --- | --- | --- | --- |
|  |  | **Experimental** *(from Step 2)* | **Comparator 1** |
| TIDieR – Item 1 [Brief Name] | What is the **name or phrase** that labels your intervention? | Cognitive Behavioral Therapy (CBT) | Attention Control |
| TIDieR – Exapnsion item^  [WHAT] | What are the known or hypothesized **active ingredients** of your Intervention? | Cognitive Behavioral Therapy (CBT); Content; Structure; Delivery (including workbook); Active participation; Talking; Time spent in a social situation | Talking, workbook/homework, travel, socialization time, using a device |
| TIDieR – Exapnsion item^  [WHAT] | What are the known or hypothesized **inactive ingredients** of your intervention? | Travel; Using a computer/device; ingredients of your experimental intervention? | The content: preventing cardiovascular disease - should not cover anything specifically targeted at  mental health |
| TIDieR – Item 2 [WHY] | What is the **rationale** behind your active and/or inactive ingredients? | Active: CBT is a type of talking therapy that is designed to help modify thinking patterns and change mood and behaviors. CBT is one of the most evidence-based psychological interventions for treating depression in the general population. There is limited evidence of effectiveness in people with stroke. For research purposes, it is structured over a pre-specified number of weeks, sessions of a pre-specified topic and duration and workbooks/homework for participants. CBT can be delivered via telephone, online, face-to-face; one to one or in group sessions. The duration of interaction with others might be significantly more than usual. Mixing socially with other people and the length of time spent doing this. Outcome assessments (asking about mood) may be considered an active aspect of the trial/intervention.  Inactive: Time, structure and social aspects need controlling. An exchange of utterances between two or more people without the intent of modifying thinking patterns or behaviors. | The selection of a control that has human to human interaction (presenting education on a different topic) is to determine whether the active CBT therapy had a difference between the groups or whether it was the time, structure and environmental aspects that made a difference. |
| TIDieR – Item 3 [WHAT] | What **materials** will be part of your intervention? | Workbook/homework: Can be paper-based and/or online. Computer/device: When CBT is delivered via telephone or online. Private space(s): For therapist and participant(s) regardless of whether they are conducing CBT via telephone, online or face-to-face. | Workbooks and homework will be about cardiovascular disease and other generic stroke topics that do not target mental health. Computer and private space same as the CBT group. |
| TIDieR – Item 4 [WHAT] | What **procedures** will be part of your intervention? | At time on consent, the participant selects their preferred model of intervention delivery (see below for details). The trial therapist then provides them with a timetable outlining when the sessions would occur throughout the experimental period. For those participants who select telephone/online delivery, they will be provided with a number or Zoom link to accommodate the sessions and this process will be trialed prior to the commencement of therapy. If face to face (individual or group) is selected, then participant will be shown where the treatment will occur and how they can access the building prior to experimental intervention commencing. | Same procedures as the CBT group, however the sessions will reflect the control intervention (attention control). |
| TIDieR – Item 5 [WHO PROVIDED] | **Who** will provide the intervention? | Trained, supervised, culturally competent healthcare professional (nurse, psychologist, other), who completes and passes all trial-related CBT training and assessment(s), and is willing and able to be supervised.  Training: GCP, protocol/trial procedures, CBT program manual, workbook. | Trained lay persons who complete and pass the trial-related attention control training. |
| TIDieR – Item 6 [HOW] | **How will** the intervention be provided? | All modes allowed: Telephone, online, face-to-face, group and individual as per the preference of the participant, as long as in a private space. | All modes allowed as with CBT group. |
| TIDieR – Item 7 [WHERE] | **Where** will the intervention be provided? | Office space for therapist with telephone/computer for telephone/online delivery and sufficient room to conduct one to one or small-group (up to 4 people) sessions. | Same options as CBT group. |
| TIDieR – Item 8 [WHEN and HOW MUCH] | **When and how much** of the intervention will be provided? | There will be 6 manualized CBT sessions to be completed over 12 weeks with a minimum of six days between sessions. Each session is designed to take up to 60 minutes. The shortest time the sessions may be completed is within 6 weeks, the maximum time is 12 weeks. | Same as the active intervention: There will be 6 cardiovascular health sessions to be completed over 12 weeks with a minimum of six days between sessions. Each session is designed to take up to 60 minutes. The shortest time the sessions may be completed is within 6 weeks, the maximum time is 12weeks. |
| TIDieR – Item 9 [TAILORING] | **How will** the intervention be tailored? | No tailoring | No tailoring |
| TIDieR – Item 11 [HOW WELL - PLANNED] | **How well will** the intervention be provided? | Sessions will be recorded and assessed for adherence to the protocol by the delegated adherence to the protocol by the delegated investigator. When therapists are found to deviate from the protocol, they will be required to attend re-training with the delegated investigator, which may be tailored specifically to the deviation rather than repeat the full training if appropriate. They will be required to pass a retraining-related assessment. | Same as the CBT group |
| **Note that TIDieR Item 10 [MODIFICATIONS] and TIDieR Item 12 [HOW WELL – ACTUAL] are not included as trial intervention is yet to be administered.*  *^As described in the main paper, expansion of the WHAT section to include active and inactive ingredients.* | | | |

| **Congratulations, you have completed all the items in the tool!**  *If you choose the "Save & Return Later" button, you will be given a randomly generated access code to re-enter the tool and revise your answers.*  *If you chose the "Submit" button, you will be given the option to download a PDF document with your answers AND a randomly-generate access code to re-enter the tool and revise your answers.* |
| --- |

**SRRR CONtrol comparator DeSIGN [CONSIGN] Tool**

Exemplar 3 Clinical language trial

Phase IIb clinical trial of a hypothetical behavioral (non-pharmacological) language intervention to improve naming in the chronic recovery epoch post-stroke.

Rationale: Emerging evidence has highlighted the importance of intensive language stimulation and therapy tasks, particularly in the chronic stage. Computer based language tasks offer an opportunity to deliver therapist-led, home-based self-directed engagement with therapeutic tasks which can be adjusted to fit around an individual’s fatigue levels, commitments and lifestyle. A common challenge during design of trials conducted in the chronic phase post-stroke is that usual care can be no therapy. This trial was designed to demonstrate the use of control comparator groups of a ‘no treatment’ and ‘placebo’ computer treatment with non-linguistic stimuli.

*Please note that the output below may appear slightly different to the PDF you can download from REDCap. The questions are consistent, but small changes have been made to formatting for publication purposes.*

| Are you undertaking a trial? | ⭘ No  ⭘ Yes, a preclinical trial  **⮾ Yes, a clinical trial** |
| --- | --- |

| **Step 1: Research question(s) and hypotheses**  *The research question is a critical motivator of control intervention design. In this section, you will explicitly specify the research question (or research aim, depending on how you prefer to phrase it) and hypotheses you intend to evaluate. At the end of Step 1, you will decide to proceed or exit the tool.* |
| --- |

| What is your **research question**(s)? | Does U-CAN treatment (a novel Computer Aphasia Naming treatment) result in better outcomes (improved naming in conversation) than no treatment or computer treatment with non-linguistic stimuli? |
| --- | --- |
| What is your **primary hypothesis**? | Practice on semantic and phonologic aspects of words, as provided in the U-CAN treatment, is necessary to improve naming in conversation compared to no treatment or computer treatment with non-linguistic stimuli. |
| Do you have additional hypotheses? | **⮾ No**  ⭘ Yes |

| **You may not need this tool if:**  *Your study is PURELY focused on feasibility. This means your question/aim is to examine the ability to conduct your study (e.g. recruitment, ability to deliver the intervention, retention, etc.) and NOT to demonstrate a causal link between the intervention and the outcome. If this is true, you may not need this tool as it predominantly addresses the selection of a control condition for causal studies. You plan to use an Objective Performance Criteria (OPC). An OPC is a numerical target value derived from historical data and may be used in a dichotomous (pass/fail) manner for review and comparison of safety, or effectiveness endpoints. If this is true, you may not need this tool as your comparison will be made against an OPC rather that a control condition. To learn more: Objective performance criteria, Section 7.6.1.*  **You need this tool if:**  *You plan to explicitly evaluate a causal link between the experimental intervention and the outcome of interest. This means you are trying to test if the intervention leads to the outcome of interest. This includes the examination of safety, dose, efficacy, effectiveness, or a combination thereof. This may also include an examination of feasibility of the control intervention(s). If this is true, continue completing this tool because you will need to select a control condition.* |
| --- |

| Do you wish to continue with this tool? | ⭘ No  **⮾ Yes** |
| --- | --- |

**Step 2: Experimental Intervention Description**

*There are elements of your experimental intervention that can impact the selection of your control intervention. The TIDieR is an established tool to guide the reporting of intervention content. In this step, you will work through the elements of your experimental intervention using a modified version of the TIDieR.*

| **Corresponding TIDieR Item** | **Question** | **Experimental Intervention Description** |
| --- | --- | --- |
| TIDieR – Item 1  [Brief Name] | What is the **name or phrase** that labels your experimental intervention? | U-CAN, a Computer-based Aphasia Naming treatment |
| TIDieR – Expansion item^  [WHAT] | What are the known or hypothesized **active ingredients** of your experimental Intervention? | 1) Semantic and phonologic features of the stimuli (pictures); 2) given at the demonstrated dose and difficulty level of task relative to aphasia severity; 3) Functional relevance of stimuli; 4) Attention associated with the general trial processes (e.g., baseline and post-treatment assessments) and intervention-related contact points. |
| TIDieR – Expansion item^  [WHAT] | What are the known or hypothesized **inactive ingredients** of your experimental intervention? | The mode of the presentation via phone, iPad, laptop, or desktop should not affect outcomes. |
| TIDieR – Item 2  [WHY] | What is the **rationale** behind your active and/or inactive ingredients? | 1) The semantic and phonologic nature of the stimuli (pictures) might be necessary to improve naming.  2) These stimuli need to be provided at sufficient doses to improve brain circuitry and naming behaviour.  3) Stimuli need to be graded to the severity of the individual's aphasia in order to engage the individual and facilitate learning.  4) Stimuli should be functionally relevant to the participant to engage the individual and facilitate generalization to real-life communication needs.  5) Human to human attention may provide increased opportunities for language stimulation and facilitate generalization to daily conversations. |
| TIDieR – Item 3  [WHAT] | What **materials** will be part of your experimental interventions? | Computer presentation of picture alone; then computer presentation of picture plus initial phoneme (sound) spoken; then picture plus initial grapheme (letter); then whole word written and spoken. The participant attempts to state the name of the picture with each presentation by the computer. Picture bank will include items with cultural and linguistic relevance to a wide-range of individuals and be customisable by the experimenter. |
| TIDieR – Item 4  [WHAT] | What **procedures** will be part of your experimental interventions? | At the time of consent, the trial staff will discuss the participants' preferred device option (e.g., phone vs laptop) and provide aphasia friendly how to guides regarding access to the experimental program based on the device option selected. This information will also be provided to carer/family member if able. The trial staff will check that the participant is able to access the program prior to experimental intervention commencing and will be available to support the participant to work through any IT concerns arise throughout the experimental period. |
| TIDieR – Item 5  [WHO PROVIDED] | **Who** will provide the experimental intervention? | A computer provides all the naming stimuli. A speech- language pathologist, with expertise in aphasia, supported communication, computer technology, and clinical research training sets up the equipment and provides training, and checks adherence remotely. |
| TIDieR – Item 6  [HOW] | **How will** the experimental intervention be provided? | All components are provided one-on-one. The computer training is done individually and independently by the participant. The intervention related contact points (e.g., set-up and training) are done with the speech-language pathologist. |
| TIDieR – Item 7  [WHERE] | **Where** will the experimental intervention be provided? | Within participant homes. Adherence monitoring is done remotely. |
| TIDieR – Item 8  [WHEN and HOW MUCH] | **When and how much** of the experimental intervention will be provided? | Computer training will be 2 x 30 min sessions/day, 6 days a week, for 6 weeks, which pilot/feasibility data suggests may be a sufficient dose to drive change. Intervention-related contact points with the speech-language pathologist will occur initially during computer set-up and training on U-CAN treatment. Decreased adherence will trigger a phone contact to the participant. |
| TIDieR – Item 9  [TAILORING] | **How will** the experimental intervention be tailored? | Stimuli will be tailored by personal relevance, language complexity, and aphasia severity. Relevance will be determined by the participants choosing words they want to practice. Initially, tailoring to aphasia severity will be done based on a pre-determined baseline measure of language ability. Level of language difficulty will be progressed automatically within the computer program. Intervention-related contact points by the speech language pathologist (SLP) will be monitored randomly to ensure fidelity i.e. they are tailored to the participant's communication level with the use of supported communication techniques and focused only on information about the intervention procedures. |
| TIDieR – Item 11  [HOW WELL - PLANNED] | **How well will** the experimental intervention be provided? | Use of the computer ensures fidelity of treatment presentation. The computer program will collect adherence data. These data will be reviewed remotely on a daily basis. If a daily session is missed, there will be a phone call to the participant. Participants must complete 85% of all U-CAN sessions to be considered adherent. |
| **Note that TIDieR Item 10 [MODIFICATIONS] and TIDieR Item 12 [HOW WELL – ACTUAL] are not included as trial intervention is yet to be administered.*  *^As described in the main paper, expansion of the WHAT section to include active and inactive ingredients.* | | |

| **Step 3. Consideration of comparator condition options**  *Now that you have information about your trial question(s), hypotheses, and experimental intervention, we will probe deeper into potential comparator conditions.*  *As you are explicitly comparing a control condition, you need to select the best control type. As you hope to demonstrate that the health outcome is due to the experimental intervention (as opposed to many other alternative explanations), you must explicitly exclude other alternative explanations.*  *This can be achieved by ensuring that the comparator condition is as similar as possible to the experimental condition in all aspects except the active ingredients.*  *In this step, you will consider various comparator options and if or how they might be suitable for your trial.* |
| --- |

| Is your comparator: | **⮾ at the group level (most common choice)**  ⭘ at the individual level (e.g., matching)  ⭘ within participant* (the participant is their own control)  *The “within participant” option is suitable when there is no underlying trend over time |
| --- | --- |

| *Now that you have information about your trial question(s), hypotheses, and experimental intervention, we will probe deeper into potential comparator conditions.*  *You may already be considering several comparator conditions. The tool outlines a range of comparator conditions available, using 11+ different labels. A definition of each type of comparator, along with when it may or may not be useful is provided to help you narrow your selection.*  *After going through this information, you may select multiple options for what you are considering. Note that some options are typically used as additional descriptors of other options. For example, protocolized + usual care, would indicate a comparator group that gets usual care that follows a specific, agreed upon trial protocol.*  *See Table 2 in the main paper for complete information.* |
| --- |

| Which comparators do you want to consider: | **⮾ Placebo / vehicle**  ⭘ Sham  **⮾** **No training / No intervention**  ⭘ Usual care  ⭘ Protocolized  ⭘ Guideline based  ⭘ Dose-matched / dose equivalent  **⮾** **Waitlist / delayed treatment**  **⮾** **Attention only**  ⭘ Active  ⭘ Historical |
| --- | --- |
| What is your working preferred control comparator(s)? | Placebo, attention control where participants do computer training with non-linguistic stimuli and have interactions related to the intervention procedures with trial staff (trained SLP). This control supports intervention-related contact points (e.g. computer set-up / training) to be similar to those provided in the U-CAN group.  No-training wait-list with delayed access to treatment control where participants receive no treatment during the delay allows for a control that may be similar to what is offered to many in the chronic phase of stroke recovery i.e., no therapy |
| Does the comparator(s) you selected require you to modify your research question or hypotheses? | ⭘ Yes  **⮾ No** |

| **Step 4. Threats to internal validity**  *Now that you have information about your trial question(s), hypotheses, and experimental intervention, we will probe deeper into potential comparator conditions.*  *As you are explicitly comparing an experimental intervention to a control condition in order to evaluate a causal link, you need to carefully anticipate possible, alternative explanations for the health outcome, other than the experimental intervention. Alternative explanations are important as they can threaten the internal validity of your trial. That is why they are termed threats. Alternate explanations may occur before the trial begins or during the conduct of the trial. Considering all possible alternative explanations now means the comparator for the trial can be designed to minimize potential threats using various strategies.*  *Designing an appropriate control group mitigates some of the threats to internal validity (so called single group threats) but some other threats (so called multiple group threats) may remain even in presence of an appropriate design. Therefore, in this step, you will consider how various common threats to stroke recovery and rehabilitation trials may or may not impact your control group. Here, the intentional focus is on threats that could arise from inadequate comparator selection. We have not included every possible threat to the trial itself. This will help you develop a working list for further discussion with your trial team, including a statistician.*  *See Figure 1 in the main paper for complete information.* |
| --- |

| Is **spontaneous recovery** a threat for your trial? | **⮾ Yes**  ⭘ No |
| --- | --- |
| *What strategies will you use to mitigate spontaneous recovery during the intervention?* | Participants are later after stroke but could still be gaining language function. Randomisation stratified based on time post stroke would help to mitigate possible effect. |
| Is an **unacceptable or uncredible comparator selection** a threat to your trial? | **⮾ Yes**  ⭘ No |
| *What strategies will you use to mitigate an unacceptable or uncredible compactor intervention?* | Careful selection of the design of the comparator group; limiting communication opportunities with the research clinician in each group to only those required for trial procedures. |
| Is **increased withdrawal from the control group** because of randomization a threat for your trial? | **⮾ Yes**  ⭘ No |
| *What strategies will you use to mitigate participant withdrawal from the control group?* | Both groups receive some computer practice – they are not informed prior to the start of the study that one computer treatment is active and the placebo computer activity is not. The active computer practice is offered to participants after completion of the placebo computer practice. |
| Is not knowing or **not controlling the content of the comparator** group a threat to your trial? | ⭘ Yes  **⮾ No** |
| *What strategies will you use to mitigate the unknown or uncontrolled comparator intervention?* | Not applicable |
| Does the **act of coming into a clinic** a threat for your trial? | ⭘ Yes  **⮾ No** |
| *What strategies will you use to mitigate increased participant activity due to simply coming into the clinic?* | In this case, participants are not coming into the clinic but are doing the intervention in the home. Both groups will have a similar number and duration of trial related contact points. |
| What **additional threat(s)** do you have and what strategies will you use to mitigate those threats? | A major threat to internal validity is severity of aphasia and other co-morbid stroke sequelae eg depression, which may impact recovery demonstrated. These will be mitigated by: 1) enrolment criteria to ensure participants can do the easiest form of the treatment and won't have a ceiling effect; and 2) stratified randomisation by Western Aphasia Battery-Revised Aphasia Quotient (WAB-R AQ). |

| **You have identified the following threats and strategies to internal validity:**   - Spontaneous recovery: Participants are later after stroke but could still be gaining language function. Stratified Randomisation to group should mitigate those effects. - Not credible or acceptable comparator: Careful selection of the design of the comparator group; limiting communication opportunities with the research clinician in each group to only those required for trial procedures. - Increased comparator group withdrawal: Both groups receive some computer practice – they are not informed prior to the start of the study that one computer treatment is active and the other computer activity is not. The other computer practice is offered to participants after completion of the first type of computer practice. - Unknown or uncontrolled comparator intervention: Not applicable. - Increased activity from coming into clinic*:* In this case, participants are not coming into the clinic but are doing the intervention in the home. Both groups will have the similar number and duration of trial related contact points. - Additional threats and strategies: A major threat to the overall trial design is severity of aphasia and other co-morbid stroke sequelae. These will be mitigated by: 1) enrolment criteria to ensure participants can do the easiest form of the treatment and won't have a ceiling effect; 2) stratified randomization by the Western Aphasia Battery – Revised Aphasia Quotient; 3) treatment that is individualized to severity level and progressed according to rules; 4) allowing the option to do more frequent but shorter sessions/day if 30 min is too long; and 5) adaptations for computer use for upper limb hemiparesis and visual impairments as needed. |
| --- |

| Do any of these **threats** require you to **modify your research question or hypotheses?** | ⭘ Yes  **⮾ No** |
| --- | --- |
| Do any of these **threats** require you to **modify your comparator condition selection(s)?** | ⭘ Yes  **⮾ No** |
| Your preferred control: | **Attention control** where participants do computer training with non-linguistic stimuli and have interactions related to the intervention procedures with trial staff (trained SLP). These intervention-related contact points (e.g. computer set-up / training) are similar to those provided in the U-CAN group.  A **wait-list delayed treatment** control where participants receive no other treatment during the delay. |

| **Step 5: Feasibility Considerations**  *You are now ready to ponder if the design and comparator conditions you have selected will be feasible to execute in your trial. In this step, you will be asked how various potential feasibility considerations may prompt you to reconsider your selections.* |
| --- |

| Do you have feasibility considerations related to **access to participants**? | **⮾ Yes**  ⭘ No |
| --- | --- |
| *How does considerations due to access to participants impact your prior selections in this tool?* | The waitlist/delayed treatment group will maximize the number of participants who eventually receive the U-CAN treatment. |
| Do you have feasibility considerations related to **funding**? | ⭘ Yes  **⮾ No** |
| *How does considerations due to funding impact your prior selections in this tool?* | Not applicable |
| Do you have feasibility considerations related to the **trial sponsor**? | ⭘ Yes  **⮾ No** |
| *How does considerations due to funding impact your prior selections in this tool?* | Not applicable |
| Do you have feasibility considerations related to **regulatory policies**? | ⭘ Yes  **⮾ No** |
| *How does considerations due to regulatory policies impact your prior selections in this tool?* | Not applicable |
| Do you have any **additional feasibility** considerations? | ⭘ Yes  **⮾ No** |

| **You have identified the following feasibility concerns:**   - Access issues: The waitlist/delayed treatment group will maximize the number of participants who eventually receive the U-CAN treatment. - Funding issues: Not applicable - Sponsor issues: Not applicable - Regulatory issues: Not applicable - Other feasibility issues: Not applicable |
| --- |

| Do any of your feasibility considerations require you to **modify your comparator** condition selection(s)? | ⭘ Yes  **⮾ No**  *Waitlist control was already identified as a potential comparator, but the feasibility considerations confirmed that it was an appropriate choice.* |
| --- | --- |

*Explanation: When the investigative team used the tool the first time through, they realized that they would be limited by the number of participants available. Thus, they chose to have a wait-list control to maximize the number of participants receiving the experimental intervention for feasibility reasons.*

| **Step 6: Comparator TIDieR(s)**  *You now have information about your research question, hypothesis, experimental intervention, comparator condition(s), threats and strategies to mitigate identified threats, and any adjustments for feasibility.*  *In this step, you will be asked to complete the TIDieR for your comparator condition(s). Many studies have a single comparator condition, but some research questions and hypothesis will require multiple comparator conditions. Preclinical studies often have multiple comparators to probe the biological mechanisms along with causality.  This tool allows for up to 6 comparator conditions.  If you are doing a human clinical trial and you have more than 3 comparator conditions, go back and reconsider your research question and hypotheses, in consultation with your trial design expert.* |
| --- |

| **How many** comparator conditions will you have? | ⭘ 1  **⮾ 2**  ⭘ 3 |
| --- | --- |

| **Corresponding TIDieR Item** | **Question** | **Intervention Description** | | |
| --- | --- | --- | --- | --- |
|  |  | **Experimental** *(from Step 2)* | **Comparator 1** | **Comparator 2** |
| TIDieR – Item 1 [Brief Name] | What is the **name or phrase** that labels your intervention? | U-CAN, a Computer-based Aphasia Naming treatment | Computer-based non-verbal visual task (attention control) | Waitlist control |
| TIDieR – Expansion item^  [WHAT] | What are the known or hypothesized **active ingredients** of your Intervention? | 1) Semantic and phonologic features of the stimuli (pictures) given at a sufficient dose and difficulty level of task relative to aphasia severity; 2) Functional relevance of stimuli; 3) Attention associated with the general trial processes (e.g., baseline and post-treatment assessments) and intervention-related contact points (e.g., intervention training). | Attention associated with the general trial processes (e.g., baseline and post-treatment assessments) and intervention-related contact points (e.g., intervention training). | None.  However, we note that individuals in this group will receive trial procedures such as baseline and post-treatment assessments. |
| TIDieR – Expansion item^  [WHAT] | What are the known or hypothesized **inactive ingredients** of your intervention? | The mode of the presentation is via phone, iPad, laptop, or desktop should not affect outcomes. | Non-verbal visual stimuli.  Mode of presentation via phone, iPAD, laptop, or desktop should not affect outcomes. | Participants will not receive any training /intervention - therefore no active/inactive ingredients are hypothesised or known. |
| TIDieR – Item 2 [WHY] | What is the **rationale** behind your active and/or inactive ingredients? | 1) The semantic and phonologic nature of the stimuli (pictures) might be necessary to improve naming.  2) These stimuli need to be provided at sufficient doses to improve brain circuitry and naming behaviour.  3) Stimuli need to be graded to the severity of the individual's aphasia to engage the individual and facilitate learning.  4) Stimuli should be functionally relevant to the participant to engage the individual and facilitate generalisation to real-life communication needs.  5) Human to human attention may provide increased opportunities for language stimulation and facilitate generalisation to daily conversations. | The human-to-human attention related to general trial processes may increase language stimulation opportunities.  The rationale for selecting a non-verbal visual stimuli was to reduce the chances of participants incidentally practicing naming tasks. | Participants will not receive any training /intervention - therefore no active/inactive ingredients are hypothesised or known. |
| TIDieR – Item 3 [WHAT] | What **materials** will be part of your intervention? | Computer presentation of picture alone; then computer presentation of picture plus initial phoneme (sound) spoken; then picture plus initial grapheme (letter); then whole word written and spoken. The participant attempts to state the name of the picture with each presentation by the computer. Picture bank will include items with cultural and linguistic relevance to a wide-range of individuals and be customisable by the experimenter. | Computer presentation of random pictures (shapes, colours) that do not stimulate naming. Participants match shapes and colours, with no overt naming response. | None. |
| TIDieR – Item 4 [WHAT] | What **procedures** will be part of your intervention? | At the time of consent, the trial staff will discuss the participants' preferred device option (e.g., phone vs laptop) and provide aphasia friendly how to guides regarding access to the experimental program based on the device option selected. This information will also be provided to carer/family member if able. The trial staff will check that the participant is able to access the program prior to experimental intervention commencing and will be available to support the participant to work through any IT concerns arise throughout the experimental period. | Same procedures as the experimental TIDieR, however the guides will reflect access to the control intervention. | None |
| TIDieR – Item 5 [WHO PROVIDED] | **Who** will provide the intervention? | A computer provides all the naming stimuli. A speech- language pathologist, with expertise in aphasia, supported communication, computer technology, and clinical research training sets up the equipment and provides training, and checks adherence remotely. | A computer provides the picture (shapes, colours) stimuli. A speech- language pathologist, with expertise in aphasia, supported communication, the computer technology, and with clinical research training sets up the equipment and provides training, and checks adherence remotely. | Not applicable |
| TIDieR – Item 6 [HOW] | **How will** the intervention be provided? | All components are provided one-on-one. The computer training is done individually and independently by the participant. The intervention related contact points (e.g., set-up and training) are done with the speech-language pathologist. | All components are provided one-on-one. The computer training is done individually and independently by the participant. The intervention related contact points (e.g., set-up and training) are done with the speech-language pathologist. | Not applicable |
| TIDieR – Item 7 [WHERE] | **Where** will the intervention be provided? | Within participant homes. Adherence monitoring is done remotely. | Within participant homes. Adherence monitoring is done remotely. | Not applicable |
| TIDieR – Item 8 [WHEN and HOW MUCH] | **When and how much** of the intervention will be provided? | Computer training will be 2 x 30 min sessions/day, 6 days a week, for 6 weeks, which pilot/feasibility data suggests may be a sufficient dose to drive change. Intervention-related contact points with the speech-language pathologist will occur initially during computer set-up and training on U-CAN treatment. Decreased adherence will trigger a phone contact to the participant. | Computer training will be 2 x 30 min sessions/day, 6 days a week, for 6 weeks. Intervention-related contact points with the speech-language pathologist will occur initially during computer set-up and training on the U-CAN treatment. Decreased adherence will trigger a phone contact to the participant. | Not applicable |
| TIDieR – Item 9 [TAILORING] | **How will** the intervention be tailored? | Stimuli will be tailored by personal relevance, language complexity, and aphasia severity. Relevance will be determined by the participants choosing words they want to practice. Initially, tailoring to aphasia severity will be done based on a pre-determined baseline measure of language ability. Level of language difficulty will be progressed automatically within the computer program. Intervention-related contact points by the SLP will be monitored randomly to ensure fidelity i.e. they are tailored to the participant's communication level with the use of supported communication techniques and focused only on information about the intervention procedures. | No tailoring of the computer stimuli. Task difficulty (e.g. number of stimuli presented) changes automatically based on performance accuracy. Intervention-related contact points by the SLP will be monitored randomly to ensure fidelity i.e. they are tailored to the participant's communication level with the use of supported communication techniques and focused only on information about the intervention procedures. | Not applicable |
| TIDieR – Item 11 [HOW WELL - PLANNED] | **How well will** the intervention be provided? | Use of the computer ensures fidelity of treatment presentation. The computer program will collect adherence data. These data will be reviewed remotely on a daily basis. If a daily session is missed, there will be a phone call to the participant. Participants must complete 85% of all U-CAN sessions to be considered adherent. | Use of the computer ensures fidelity of stimuli presentation. The computer program will collect adherence data. These data will be reviewed remotely on a daily basis. If a daily session is missed, there will be a phone call to the participant. Participants must complete 85% of all computer sessions to be considered adherent. | Not applicable |
| **Note that TIDieR Item 10 [MODIFICATIONS] and TIDieR Item 12 [HOW WELL – ACTUAL] are not included as trial intervention is yet to be administered.*  *^As described in the main paper, expansion of the WHAT section to include active and inactive ingredients.* | | | | |

| **Congratulations, you have completed all the items in the tool!**  *If you choose the "Save & Return Later" button, you will be given a randomly generated access code to re-enter the tool and revise your answers.*  *If you chose the "Submit" button, you will be given the option to download a PDF document with your answers AND a randomly-generate access code to re-enter the tool and revise your answers.* |
| --- |

**SRRR CONtrol comparator DeSIGN [CONSIGN] Tool**

## Exemplar 4 Clinical pharmacological and exercise trial

Phase III clinical trial of a combined hypothetical pharmacological and behavioral intervention to improve gait in the early subacute recovery epoch.

Rationale: It is increasingly recognised that drug or neuromodulation approaches will work best alongside high quality, or perhaps bespoke, rehabilitation therapy in post stroke recovery. This poses numerous challenges for comparator selection. In addition, investigators may wish to maximise efficiency by incorporating different interventions into a trial protocol. This exemplar aims to highlight how the tool might support control comparator design given these considerations.

*Please note that the output below may appear slightly different to the PDF you can download from REDCap. The questions are consistent, but small changes have been made to formatting for publication purposes.*

| Are you undertaking a trial? | ⭘ No  ⭘ Yes, a preclinical trial  **⮾ Yes, a clinical trial** |
| --- | --- |

| **Step 1: Research question(s) and hypotheses**  *The research question is a critical motivator of control intervention design. In this section, you will explicitly specify the research question (or research aim, depending on how you prefer to phrase it) and hypotheses you intend to evaluate. At the end of Step 1, you will decide to proceed or exit the tool.* |
| --- |

| What is your **research question**(s)? | Does the combination of Recoverall and Gaitercise with usual care improve gait in the early subacute recovery epoch post-stroke more than Recoverall with usual care or placebo with usual care or usual care alone?  **Note that Gaitercise has previously been proven to be effective when compared to usual care.* |
| --- | --- |
| What is your **primary hypothesis**? | The combined effects of Recoverall and Gaitercise on post-stroke gait are additive, producing better outcomes than Recoverall or usual care alone. |
| Do you have additional hypotheses? | ⭘ No  **⮾ Yes** |
|  | The individual effects of Recoverall will produce better post-stroke gait outcomes than usual care. |

| **You may not need this tool if:**  *Your study is PURELY focused on feasibility. This means your question/aim is to examine the ability to conduct your study (e.g. recruitment, ability to deliver the intervention, retention, etc.) and NOT to demonstrate a causal link between the intervention and the outcome. If this is true, you may not need this tool as it predominantly addresses the selection of a control condition for causal studies. You plan to use an Objective Performance Criteria (OPC). An OPC is a numerical target value derived from historical data and may be used in a dichotomous (pass/fail) manner for review and comparison of safety, or effectiveness endpoints. If this is true, you may not need this tool as your comparison will be made against an OPC rather that a control condition. To learn more: Objective performance criteria, Section 7.6.1.*  **You need this tool if:**  *You plan to explicitly* ***evaluate a causal link between the experimental intervention and the outcome of interest.*** *This means you are trying to test if the intervention leads to the outcome of interest. This includes the examination of safety, dose, efficacy, effectiveness, or a combination thereof. This may also include an examination of feasibility of the control intervention(s). If this is true, continue completing this tool because you will need to select a control condition.* |
| --- |

| Do you wish to continue with this tool? | ⭘ No  **⮾ Yes** |
| --- | --- |

**Step 2: Experimental Intervention Description**

*There are elements of your experimental intervention that can impact the selection of your control intervention. The TIDieR is an established tool to guide the reporting of intervention content. In this step, you will work through the elements of your experimental intervention using a modified version of the TIDieR.*

| **Corresponding TIDieR Item** | **Question** | **Experimental Intervention Description** |
| --- | --- | --- |
| TIDieR – Item 1  [Brief Name] | What is the **name or phrase** that labels your experimental intervention? | (1) Recoverall + (2) Gaitercise |
| TIDieR – Expansion item^  [WHAT] | What are the known or hypothesized **active ingredients** of your experimental Intervention? | (1) Recoverall drug  (2) Gaitercise exercise intervention involving intensive muscular strength, walking training, motor coordination, dynamic balance training, and health-behaviour techniques to maximize daily step count.  **Note that all participants will receive usual care therapy without exercise/walking retraining.* |
| TIDieR – Expansion item^  [WHAT] | What are the known or hypothesized **inactive ingredients** of your experimental intervention? | (1) Recoverall - Other compounds (filler) in the Recoverall drug that are used to package it.  (2) Gaitercise - face to face contact with the therapist. |
| TIDieR – Item 2  [WHY] | What is the **rationale** behind your active and/or inactive ingredients? | (1) Recoverall: outcomes of early phase learning studies show the drug has signal of effect to help improve synaptic plasticity following stroke.  (2) Gaitercise: exercise intervention that has been proven to be effective in in improving gait in the early subacute recovery epoch post-stroke. |
| TIDieR – Item 3  [WHAT] | What **materials** will be part of your experimental interventions? | (1) Recoverall drug - (a) information sheet regarding potential side effects, (b) Blister pack with medication allocated as per schedule.  (2) Gaitercise - requires gym equipment such as cones, plinth, steps, weights, parallel bars. |
| TIDieR – Item 4  [WHAT] | What **procedures** will be part of your experimental intervention? | (1) Recoverall drug – Drug will be dispensed in tablet form via a Blister pack by the study pharmacist and will be provided onsite, twice daily, by nursing staff to the participants for the duration of intervention period (see below items for further details). The blister pack will be checked weekly by the study pharmacy/site PI for compliance. The study pharmacy and site PI will be available via phone to answer any questions from nursing staff or participants regarding the experimental drug.  (2) Gaitercise – Participant will be provided with a timetable of when exercise intervention sessions will occur. The therapist is responsible for negotiating these times within other usual care therapy requirements. The therapist will be responsible for collecting and returning the participant to the ward from the gym area. The timetable will be checked weekly by the site PI to ensure compliance with the intervention protocol. |
| TIDieR – Item 5  [WHO PROVIDED] | **Who** will provide the experimental intervention? | (1) Recoverall drug will be dispensed by study pharmacist trained in manufacturer processes (> 10 years clinical experience) and given by ward nursing staff (> 5 years clinical experience) trained in the administration protocol. Training session for the administration protocol involved a 60 minute training session regarding medication, schedule, side effects and administration requirements.  (2) Gaitercise training will be provided by certified practicing physical therapists (>10 years clinical experience) who are trained in the Gaitercise intervention protocol. Intervention training will include attendance at a 60 minute induction session and application of protocol to five case studies.  - Site PI available to answer any trial questions via phone or email. |
| TIDieR – Item 6  [HOW] | **How will** the experimental intervention be provided? | (1) Recoverall drug - will be provided orally in tablet form by nursing staff, in a face to face capacity as per the medication schedule.  (2) Gaitercise will be provided face-to-face, one on one with a trial therapist at the rehabilitation centre. |
| TIDieR – Item 7  [WHERE] | **Where** will the experimental intervention be provided? | Recoverall and gaitercise were given under supervision at the inpatient rehabilitation unit (hospital setting). It is a combination of private and public health care funding and has all required medication and gym equipment to administered the experimental intervention. Treatment may extend out into outpatient clinics to achieve the 6 week duration, if needed. |
| TIDieR – Item 8  [WHEN and HOW MUCH] | **When and how much** of the experimental intervention will be provided? | (1) Recoverall drug - 1 x tablet will be delivered twice daily for first 6 weeks after enrolment.  (2) Gaitercise will be delivered 1 x 60 minute session, 5 days per week, for 6 weeks after trial enrolment. The first 10 minutes of session will involvement warm up exercises. The next 45 minutes will involve moderate intensity (measured by Borg rating of perceived exertion scale) multi modal exercises. The final 5 minutes will be a cool down exercise. |
| TIDieR – Item 9  [TAILORING] | **How will** the experimental intervention be tailored? | (1) Recoverall dose will not change over the course of 6 weeks  (2) Gaitercise - will be tailored to each participant based on the trial therapist clinical reasoning (within the boundaries of trial protocol). Tailoring will involve changes to weights, repetitions, intensity measures throughout the training program based on participants progress and presentation throughout the intervention. The tailoring will be measured and reported by the trial therapists. |
| TIDieR – Item 11  [HOW WELL - PLANNED] | **How well will** the experimental intervention be provided? | (1) Recoverall will be blister packed, research coordinator will check adherence by counting unopened packaging.  (2) Therapists delivering the Gaitercise will report the number of successful and unsuccessful exercises within the intervention. One session per week will be video recorded for independent fidelity monitoring of recorded dose to actual dose. |
| **Note that TIDieR Item 10 [MODIFICATIONS] and TIDieR Item 12 [HOW WELL – ACTUAL] are not included as trial intervention is yet to be administered.*  *^As described in the main paper, expansion of the WHAT section to include active and inactive ingredients.* | | |

| **Step 3. Consideration of comparator condition options**  *Now that you have information about your trial question(s), hypotheses, and experimental intervention, we will probe deeper into potential comparator conditions.*  *As you are explicitly comparing a control condition, you need to select the type of control that might be best. As you hope to demonstrate that the health outcome is due to the experimental intervention (as opposed to many other, alternative explanations), you must explicitly exclude other alternative explanations.*  *This can be achieved by making sure that the comparator condition is as similar as possible to the experimental condition in all aspects except the active ingredients. In this step, you will consider various comparator options and if or how they might be suitable for your trial.* |
| --- |

| Is your comparator: | **⮾ at the group level (most common choice)**  ⭘ at the individual level (e.g., matching)  ⭘ within participant* (the participant is their own control)  *The “within participant” option is suitable when there is no underlying trend over time |
| --- | --- |

| *Now that you have information about your trial question(s), hypotheses, and experimental intervention, we will probe deeper into potential comparator conditions.*  *You may already be considering several comparator conditions. The tool outlines a range of comparator conditions available, using 11+ different labels. A definition of each type of comparator, along with when it may or may not be useful is provided to help you narrow your selection.*  *After going through this information, you may select multiple options for what you are considering. Note that some options are typically used as additional descriptors of other options. For example, protocolized + usual care, would indicate a comparator group that gets usual care that follows a specific, agreed upon trial protocol.*  *See Table 2 in the main paper for complete information.* |
| --- |

| Which comparators do you want to consider: | **⮾ Placebo**  ⭘ Vehicle  ⭘ Sham  ⭘ No training / No intervention  **⮾ Usual care**  ⭘ Protocolized  ⭘ Guideline based  ⭘ Dose-matched / dose equivalent  ⭘ Waitlist / delayed treatment  ⭘ Attention only  **⮾ Active**  ⭘ Historical |
| --- | --- |
| What is your working preferred control comparator(s)? | 3 comparative groups to fully evaluate hypotheses:  (1) Recoverall + Usual care  (2) Placebo + Usual care  (3) Usual Care alone |
| Does the comparator(s) you selected require you to modify your research question or hypotheses? | ⭘ Yes  **⮾ No** |

| **Step 4. Threats to internal validity**  *Now that you have information about your trial question(s), hypotheses, and experimental intervention, we will probe deeper into potential comparator conditions.*  *As you are explicitly comparing an experimental intervention to a control condition in order to evaluate a causal link, you need to carefully anticipate possible, alternative explanations for the health outcome, other than the experimental intervention. Alternative explanations are important as they can threaten the internal validity of your trial. That is why they are termed threats. Alternate explanations may occur before the trial begins or during the conduct of the trial. Considering all possible alternative explanations now means the comparator for the trial can be designed to minimize potential threats using various strategies.*  *Designing an appropriate control group mitigates some of the threats to internal validity (so called single group threats) but some other threats (so called multiple group threats) may remain even in presence of an appropriate design. Therefore, in this step, you will consider how various common threats to stroke recovery and rehabilitation trials may or may not impact your control group. Here, the intentional focus is on threats that could arise from inadequate comparator selection. We have not included every possible threat to the trial itself. This will help you develop a working list for further discussion with your trial team, including a statistician.*  *See Figure 1 in the main paper for more information.* |
| --- |

| Is **spontaneous recovery** a threat for your trial? | **⮾ Yes**  ⭘ No |
| --- | --- |
| *What strategies will you use to mitigate spontaneous recovery during the intervention?* | Serial measurements to produce individual trajectories over time. All participants will be recruited within one week of stroke onset. |
| Is an **unacceptable or uncredible comparator selection** a threat to your trial? | **⮾ Yes**  ⭘ No |
| *What strategies will you use to mitigate an unacceptable or uncredible compactor intervention?* | Offer all participants Gaitercise at end of blinded follow up if they still have gait issues. |
| Is **increased withdrawal from the control group** because of randomization a threat for your trial? | **⮾ Yes**  ⭘ No |
| *What strategies will you use to mitigate participant withdrawal from the control group?* | Offer all participants Gaitercise at end of blinded follow up if they still have gait issues. |
| Is not knowing or **not controlling the content of the comparator** group a threat to your trial? | **⮾ Yes**  ⭘ No |
| *What strategies will you use to mitigate the unknown or uncontrolled comparator intervention?* | Generate a detailed reporting and fidelity plan for the groups receiving usual care. This plan will include the some videoing of treatment as planned for the experimental intervention and also recording of dose by usual care therapist for each participant. |
| Does the **act of coming into a clinic** a threat for your trial? | ⭘ Yes  **⮾ No** |
| *What strategies will you use to mitigate increased participant activity due to simply coming into the clinic?* | Not applicable. |
| What **additional threat(s)** do you have and what strategies will you use to mitigate those threats? | Loss to follow-up as the control group was not seen to benefit participants. Seek consent at enrolment to contact their general practitioner/ relatives to ascertain status if lost to follow up. |

| **You have identified the following threats and strategies to internal validity:**   - Spontaneous recovery: Serial measurements to produce individual trajectories over time. All participants will be recruited within one week of stroke onset, so chances of spontaneous are presumed equal. - Not credible or acceptable comparator: Offer all participants Gaitercise at end of blinded follow up if they still have gait issues. - Increased comparator group withdrawal: Offer all participants Gaitercise at end of blinded follow up if they still have gait issues. - Unknown or uncontrolled comparator intervention: Generate a detailed reporting and fidelity plan for the groups receiving usual care. This plan will include the some videoing of treatment as planned for the experimental intervention and also recording of dose by usual care therapist for each participant. - Increased activity from coming into clinic*:* Not applicable. - Additional threats and strategies: Loss to follow-up as the control group was not seen to benefit participants. Seek consent at enrolment to contact G.P / relatives to ascertain status if lost to follow up. |
| --- |

| Do any of these **threats** require you to **modify your research question or hypotheses?** | ⭘ Yes  **⮾ No** |
| --- | --- |
| Do any of these **threats** require you to **modify your comparator condition selection(s)?** | ⭘ Yes  **⮾ No** |
| Your preferred control: | (1) Recoverall + Usual care  (2) Placebo + Usual care  (3) Usual Care alone |

| **Step 5: Feasibility Considerations**  *You are now ready to ponder if the design and comparator conditions you have selected will be feasible to execute in your trial. In this step, you will be asked how various potential feasibility considerations may prompt you to reconsider your selections.* |
| --- |

| Do you have feasibility considerations related to **access to participants**? | **⮾ Yes**  ⭘ No |
| --- | --- |
| *How does considerations due to access to participants impact your prior selections in this tool?* | Eligibility criteria require people to be able to perform gait exercises early after stroke which may impact eligible participant pool (e.g., those with more severe post-stroke impairments may not be eligible for the trial). May need more trial sites than planned to reach target numbers. More sites result in more potential variability in usual care. |
| Do you have feasibility considerations related to **funding**? | **⮾ Yes**  ⭘ No |
| *How does considerations due to funding impact your prior selections in this tool?* | 1. Differences in Usual Care: Usual care will differ significantly across countries. Some sites with minimal rehabilitation services may argue to supplement usual care which would require extra cost to the trial.  2. As such focus only on physiotherapy/physical therapy-related usual care (not all therapy provided).  This is reflected in TIDieR usual care |
| Do you have feasibility considerations related to the **trial sponsor**? | ⭘ Yes  **⮾ No** |
| *How does considerations due to funding impact your prior selections in this tool?* | Not applicable |
| Do you have feasibility considerations related to **regulatory policies**? | **⮾ Yes**  ⭘ No |
| *How does considerations due to regulatory policies impact your prior selections in this tool?* | New drug requires approval in multiple countries to run this trial which may cause delays to start up. |
| Do you have any **additional feasibility** considerations? | ⭘ Yes  **⮾ No** |
| **You have identified the following feasibility concerns:**   - Access issues: Eligibility criteria require people to be able to perform gait exercises early after stroke which may impact eligible participant pool (e.g., those with more severe post-stroke impairments may not be eligible for the trial). May need more trial sites than planned to reach target numbers. More sites result in more potential variability in usual care. - Funding issues: 1. Differences in Usual Care: Usual care will differ significantly across countries. Some sites with minimal rehabilitation services may argue to supplement usual care which would require extra cost to the trial. 2. As such focus only on physiotherapy/physical therapy-related usual care (not all therapy provided). This is reflected in TIDieR usual care - Sponsor issues: Not applicable - Regulatory issues: New drug requires approval in multiple countries to run this trial which may cause delays to start up. - Other feasibility issues: Not applicable | |

| Do any of your feasibility considerations require you to **modify your comparator** condition selection(s)? | ⭘ Yes  **⮾ No** |
| --- | --- |

| **Step 6: Comparator TIDieR(s)**  *You now have information about your research question, hypothesis, experimental intervention, comparator condition(s), threats and strategies to mitigate identified threats, and any adjustments for feasibility.*  *In this step, you will be asked to complete the TIDieR for your comparator condition(s). Many studies have a single comparator condition, but some research questions and hypothesis will require multiple comparator conditions. Preclinical studies often have multiple comparators to probe the biological mechanisms along with causality.  This tool allows for up to 6 comparator conditions.  If you are doing a human clinical trial and you have more than 3 comparator conditions, go back and reconsider your research question and hypotheses, in consultation with your trial design expert.* |
| --- |

| **How many** comparator conditions will you have? | ⭘ 1  ⭘ 2  **⮾ 3** |
| --- | --- |

|  |  | **Intervention Description** | | | |
| --- | --- | --- | --- | --- | --- |
| **Corresponding TIDieR Item** | **Question** | **Experimental** *(from Step 2)* | **Comparator 1** | **Comparator 2** | **Comparator 3** |
| TIDieR – Item 1 [Brief Name] | What is the **name or phrase** that labels your intervention? | (1) Recoverall + (2) Gaitercise | (1) Recoverall + (2) Usual Care | (1) Placebo + (2) Usual care | Usual care alone |
| TIDieR – Expansion item^  [WHAT] | What are the known or hypothesized **active ingredients** of your Intervention? | (1) Recoverall drug – active properities.  (2) Gaitercise exercise intervention involving intensive muscular strength, walking training, motor coordination, dynamic balance training, and health-behaviour techniques to maximize daily step count.  **Note that all participants will receive usual care therapy without exercise/walking retraining.* | (1) Recoverall drug - active properties.  (2) Usual care - involves exercise intervention that can include a combination of traditional gait re-training, balance retraining, and strengthening exercises. Group sessions involved hydrotherapy and balance group. | (1) Placebo - No active ingredients identified.  (2) Usual care – see Comparator 1 TIDieR | Usual care – see Comparator 1 TIDieR |
| TIDieR – Expansion item^  [WHAT] | What are the known or hypothesized **inactive ingredients** of your intervention? | (1) Recoverall - Other compounds (filler) in the Recoverall drug that are used to package it.  (2) Gaitercise - face to face contact with the therapist. | (1) Recoverall – see experimental TIDieR  (2) Usual Care - face to face contact with the therapist. | (1) Placebo - effect of taking medication.  (2) Usual care – see Comparator 1 TIDieR | Usual care – see Comparator 1 TIDieR |
| TIDieR – Item 2 [WHY] | What is the **rationale** behind your active and/or inactive ingredients? | (1) Recoverall: outcomes of early phase learning studies show the drug has signal of effect to help improve synaptic plasticity following stroke.  (2) Gaitercise: exercise intervention that has been proven to be effective in in improving gait in the early subacute recovery epoch post-stroke. | (1) Recoverall – see experimental TIDieR  (2) The human-to-human attention from usual care therapy. | (1) Placebo – used to ensure the group differences are not related to the process of taking the medication.  (2) Usual care – see Comparator 1 TIDieR | Usual care – see Comparator 1 TIDieR |
| TIDieR – Item 3 [WHAT] | What **materials** will be part of your intervention? | (1) Recoverall drug - (a) information sheet regarding potential side effects, (b) Blister pack with medication allocated as per schedule.  (2) Gaitercise - requires gym equipment such as cones, plinth, steps, weights, parallel bars. | (1) Recoverall – see experimental TIDieR  (2) Usual care - requires gym equipment such as cones, plinth, steps, weights, parallel bars. Hydrotherapy pool, including flotation devices, pool noodles. | (1) Placebo - (a) information sheet consistent with the experimental intervention about potential side effects (b) Blister pack with medication allocated as per schedule.  (2) Usual care – see Comparator 1 TIDieR | Usual care – see Comparator 1 TIDieR |
| TIDieR – Item 4 [WHAT] | What **procedures** will be part of your intervention? | (1) Recoverall drug – Drug will be dispensed in tablet form via a Blister pack by the study pharmacist and will be provided onsite, twice daily, by nursing staff to the participants for the duration of intervention period (see below items for further details). The blister pack will be checked weekly by the study pharmacy/site PI for compliance. The study pharmacy and site PI will be available via phone to answer any questions from nursing staff or participants regarding the experimental drug.  (2) Gaitercise – Participant will be provided with a timetable of when exercise intervention sessions will occur. The therapist is responsible for negotiating these times within other usual care therapy requirements. The therapist will be responsible for collecting and returning the participant to the ward from the gym area. The timetable will be checked weekly by the site PI to ensure compliance with the intervention protocol. | (1) Recoverall – see experimental TIDieR  (2) Usual care – will be timetabled each week for the individual participant in accordance with their specific goals and needs. A porter will collect the patient from the ward and return them to the ward after therapy. A multidisciplinary planning meeting will occur weekly with the purpose to update goals and complete discharge planning. | (1) Placebo – see Experimental TIDieR for procedure details with the drug dispensed in the webster pack a placebo rather than the Recoverall drug.  (2) Usual care – see Comparator 1 TIDieR | Usual care – see Comparator 1 TIDieR |
| TIDieR – Item 5 [WHO PROVIDED] | **Who** will provide the intervention? | (1) Recoverall drug will be dispensed by study pharmacist trained in manufacturer processes (> 10 years clinical experience) and given by ward nursing staff (> 5 years clinical experience) trained in the administration protocol. Training session for the administration protocol involved a 60 minute training session regarding medication, schedule, side effects and administration requirements.  (2) Gaitercise training will be provided by certified practicing physical therapists (>10 years clinical experience) who are trained in the Gaitercise intervention protocol. Intervention training will include attendance at a 60 minute induction session and application of protocol to five case studies.  - Site PI available to answer any trial questions via phone or email. | (1) Recoverall – see experimental TIDieR  (2) Usual care will be provided by certified practicing physical therapists (no experience limits) and physical therapy assistants (no experience limits) working at the inpatient rehabilitation unit. No additional training will be provided re: provision of usual care, except for a 30 minute training session regarding how to record usual care dose in line with trial protocol. | (1) Placebo will be consistently administered as per experimental intervention.  (2) Usual care – see Comparator 1 TIDieR | Usual care – see Comparator 1 TIDieR |
| TIDieR – Item 6 [HOW] | **How will** the intervention be provided? | (1) Recoverall drug - will be provided orally in tablet form by nursing staff, in a face to face capacity as per the medication schedule.  (2) Gaitercise will be provided face-to-face, one on one with a trial therapist at the rehabilitation centre. | (1) Recoverall - see experimental TIDieR  (2) Usual care control is provided by clinical team, face to face in a combination of one-on-one and group based interventions (max of 6 people per group). | (1) Placebo - consistent with experimental intervention.  (2) Usual care – see Comparator 1 TIDieR | Usual care – see Comparator 1 TIDieR |
| TIDieR – Item 7 [WHERE] | **Where** will the intervention be provided? | Recoverall and gaitercise were given under supervision at the inpatient rehabilitation unit (hospital setting). It is a combination of private and public health care funding and has all required medication and gym equipment to administered the experimental intervention. Treatment may extend out into outpatient clinics to achieve the 6 week duration, if needed. | Recoverall + Usual Care will be delivered in the same location and with the same healthcare professionals as the experimental group. Treatment may extend out to outpatient clinics deemed appropriate by the treating team. | Placebo + Usual Care will be delivered in the same location and with the same healthcare professionals as the experimental group. Treatment may extend out to outpatient clinics deemed appropriate by the treating team. | Usual care – see Comparator 1 TIDieR |
| TIDieR – Item 8 [WHEN and HOW MUCH] | **When and how much** of the intervention will be provided? | (1) Recoverall drug - 1 x tablet will be delivered twice daily for first 6 weeks after enrolment.  (2) Gaitercise will be delivered 1x60 minute session, 5 days per week, for 6 weeks after trial enrolment. The first 10 minutes of session will involvement warm up exercises. The next 45 minutes will involve moderate intensity (measured by Borg rating of perceived exertion scale) multi modal exercises. The final 5 minutes will be a cool down exercise. | (1) Recoverall - see experimental TIDieR  (2) Usual Care will be provided in line with local hospital guidelines, trial staff will not specifically outline any requirements. | (1) Placebo - consistent with experimental intervention.  (2) Usual care – see Comparator 1 TIDieR | Usual care – see Comparator 1 TIDieR |
| TIDieR – Item 9 [TAILORING] | **How will** the intervention be tailored? | (1) Recoverall dose will not change over the course of 6 weeks  (2) Gaitercise - will be tailored to each participant based on the trial therapist clinical reasoning (within the boundaries of trial protocol). Tailoring will involve changes to weights, repetitions, intensity measures throughout the training program based on participants progress and presentation throughout the intervention. The tailoring will be measured and reported by the trial therapists. | (1) Recoverall - see experimental TIDieR  (2) Usual care will be tailored to individual needs at the discretion of the treating therapists as per usual care normally. | (1) Placebo - consistent with experimental group.  (2) Usual care – see Comparator 1 TIDieR | Usual care – see Comparator 1 TIDieR |
| TIDieR – Item 11 [HOW WELL - PLANNED] | **How well will** the intervention be provided? | (1) Recoverall will be blister packed, research coordinator will check adherence by counting unopened packaging.  (2) Therapists delivering the Gaitercise will report the number of successful and unsuccessful exercises within the intervention. One session per week will be video recorded for independent fidelity monitoring of recorded dose to actual dose. | (1) Recoverall - see experimental TIDieR  (2) Usual care - therapy will be recorded on an electronic database and 1 session every week recorded for fidelity purposes. | (1) Placebo - consistent with the experimental group.  (2) Usual care- Usual care – see Comparator 1 TIDieR | Usual care – see Comparator 1 TIDieR |
| **Note that TIDieR Item 10 [MODIFICATIONS] and TIDieR Item 12 [HOW WELL – ACTUAL] are not included as trial intervention is yet to be administered.*  *^As described in the main paper, expansion of the WHAT section to include active and inactive ingredients.* | | | | | |

| **Congratulations, you have completed all the items in the tool!**  *If you choose the "Save & Return Later" button, you will be given a randomly generated access code to re-enter the tool and revise your answers.*  *If you chose the "Submit" button, you will be given the option to download a PDF document with your answers AND a randomly-generate access code to re-enter the tool and revise your answers.* |
| --- |

# Supplemental 2: Key references with statement related to control comparator design.

1. AVERT Trial Collaboration, Bernhardt J, Langhorne P, et al. (2015). Efficacy and safety of very early mobilisation within 24 h of stroke onset (AVERT): a randomised controlled trial. *Lancet*, *386*(9988), 46-55.

*Explanation:* *An example of a Phase III trial with a usual care which had challenges associated with the intervention active ingredient (time to mobilisation). In this trial, the time to intervention (very early mobilisation) and control (usual care) were not dissimilar.*

1. Bernhardt J, Hayward KS, Dancause N, et al. (2019). A stroke recovery trial development framework: Consensus-based core recommendations from the Second Stroke Recovery and Rehabilitation Roundtable. International Journal of Stroke, 14, 792-802.

*Explanation:* *This consensus statement is from the second Stroke Recovery and Rehabilitation Roundtable (SRRR) describes key issues for the next generation of stroke recovery treatment trials and presents the Trials Development Framework (SRRR-TDF). This framework is designed to guide the GO, NO-GO decision-making process in trial development.*

1. Birkenmeier RL, Prager EM, & Lang CE. (2010). Translating animal doses of task-specific training to people with chronic stroke in 1-hour therapy sessions: a proof-of-concept study. Neurorehabil Neural Repair, 24(7), 620-635

*Explanation: Example of a Phase II feasibility trial that does not have a control group as the question is feasibility. In such examples, the CONSIGN tool is not required.*

1. Brady MC, Godwin J, Kelly H, Enderby P, Elders A, & Campbell P. (2018). Attention control comparisons with SLT for people with aphasia following stroke: methodological concerns raised following a systematic review. Clin Rehabil, 32(10), 1383-1395.

*Explanation: This review demonstrated how poor comparator choice may erode the distinction between an experimental intervention and an attention control comparator that includes highly active ingredients and resulted in a higher number of participant drop-outs from the control arm, potentially suggesting that the control intervention was unacceptable to, or less tolerated by, participants allocated to that group.*

1. Breitenstein C, Grewe T, Floel A, et al. (2017). Intensive speech and language therapy in patients with chronic aphasia after stroke: a randomised, open-label, blinded-endpoint, controlled trial in a health-care setting. Lancet, 389(10078), 1528-1538.

*Explanation: An example of a multisite trial in which people with chronic aphasia were randomized to 3 weeks of intensive speech and language therapy or 3 weeks of deferral of intensive speech and language therapy (i.e., waitlist control group). Participants in the control group received identical intensive speech and language therapy starting immediately after the 3-week waiting period. Once the control group had received the intensive speech and language therapy, participants showed similar improvements in primary and secondary language outcomes to the intervention group, thus replicating the primary treatment effect.*

1. Dawson J, Liu CY, Francisco GE, et al. (2021). Vagus nerve stimulation paired with rehabilitation for upper limb motor function after ischaemic stroke (VNS-REHAB): a randomised, blinded, pivotal, device trial. Lancet, 397(10284), 1545-1553.

*Explanation: An example of a phase III trial of an implantable nerve stimulator that was compared to sham. It would not be possible to blind the trial if control participants were not implanted with the device. And even if they were, there would be a risk to blinding if the sensation of active stimulation was not delivered to control participants (approximately half of all people can perceive VNS at the stimulation level used). Therefore, all participants were implanted with the device and were randomised to active or sham stimulation. At the start of each therapy session, all participants regardless of group allocation received a small number of active stimulations to give all participants an equal chance of perception.*

1. Food and Drug Administration: Center for Biologics Evaluation and Research & Center for Devices and Radiological Health. Design Considerations for Pivotal Clinical Investigations for Medical Devices: Guidance for Industry, Clinical Investigators, Institutional Review Boards and FDA Staff. November 7, 2013.

*Explanation: Guidance on designing clinical studies. Sections to highlight: Section 7.4 discusses “Controls in comparative effectiveness clinical outcome studies” and Section 7.6.1 discusses “Single-Group Study with Objective Performance Criterion (OPC)”*

1. Ghadessi, M., Tang, R., Zhou, J., Liu, R., Wang, C., Toyoizumi, K., Mei, C., Zhang, L., Deng, C. Q., & Beckman, R. A. (2020). A roadmap to using historical controls in clinical trials - by Drug Information Association Adaptive Design Scientific Working Group (DIA-ADSWG). Orphanet J Rare Dis, 15(1), 69.

*This paper provides a comprehensive roadmap for planning, conducting, analyzing and reporting of studies using historical controls, mainly when a randomized clinical trial is not possible.*

1. Hayward KS, Kramer SF, Dalton EJ, et al. (2021). Timing and Dose of Upper Limb Motor Intervention After Stroke: A Systematic Review. Stroke, 52(11), 3706-3717.

*Example: This review of upper limb rehabilitation and recovery trials completed in the first 6 months post stroke shows that most trials use either usual care or dose-matched usual care as the control type of choice.*

1. Hill MD, Goyal M, Menon BK, et al. Efficacy and safety of nerinetide for the treatment of acute ischaemic stroke (ESCAPE-NA1): A multicentre, double-blind, randomised controlled trial. Lancet. 2020;395:878–887.

*Explanation: The importance of choosing the correct control was demonstrated in this trial. NA1 showed lack of efficacy in the presence of thrombolysis, a treatment confound rarely assessed preclinically. Preclinical studies were positive in non-human primates (Cook et al., 2012, Nature), rats (Sun et al., 2008, Stroke), and mice (Teves et al., 2016, J Cereb Blood Flow Metab).*

1. Hoffmann TC, Glaszio PP, Boutron I, et al. (2014). Better reporting of interventions: template for intervention description and replication (TIDieR) checklist and guide. BMJ, 348, g1687.

*Explanation:* *The Template for Intervention Description and Replication (TIDieR) checklist and guide was developed to improve the completeness of reporting, and ultimately the replicability, of interventions* *(experimental and control).* *The checklist is an extension of the CONSORT 2010 statement (item 5) and the SPIRIT 2013 statement (item 11).*

1. Lohse KR, Pathania A, Wegman R, Boyd LA, & Lang CE. (2018). On the Reporting of Experimental and Control Therapies in Stroke Rehabilitation Trials: A Systematic Review. Arch Phys Med Rehabil, 99(7), 1424-1432.

*Explanation: This review of reported methods across stroke rehabilitation trials shows that published papers generally provide minimal description and referencing for control comparator conditions, making it difficult to synthesize data across trials****.***

1. Rodgers H, Bosomworth H, Krebs HI, et al. (2019). Robot assisted training for the upper limb after stroke (RATULS): a multicentre randomised controlled trial. Lancet. 24(54):1-232

*Explanation: An example of a Phase III trial of robot assisted training that was compared to dose-matched usual care and usual care. In this trial, two comparators were used in order to assess whether a) there was a specific effect of robotic therapy separate to the time and activity component of delivering the treatment (dose-matched comparator), and b) to quantify the effect of robotic therapy versus standard of care in the National Health Service UK (usual care comparator).*

1. The National Institute of Neurological Disorders and Stroke rt-PA Stroke Study Group. (1995). Tissue plasminogen activator for acute ischemic stroke. N Engl J Med. 333(24):1581-1587.

*Explanation: An example of a Phase III trial of a pharmaceutical agent for stroke recovery that is tested against a placebo only. Evaluation against a placebo is the standard to achieve regulatory approval for new agents and is a lower bar to overcome than evaluation against a comparator with a known benefit.*

1. Trochim, W. M. K. Single group threats <https://conjointly.com/kb/single-group-threats/> and Multiple group threats <https://conjointly.com/kb/multiple-group-threats/>.

*These two webpages discuss all the types of single and multiple group threats for consideration during trial design.*

1. Winstein CJ, Wolf SL, Dromerick AW, et al. (2016) Effect of a Task-Oriented Rehabilitation Program on Upper Extremity Recovery Following Motor Stroke: The ICARE Randomized Clinical Trial. JAMA*.*315(6):571-581.

*Explanation: An example of a Phase III trial that compares a novel intervention to usual care, using two comparator groups: usual care and dose-matched usual care, in order to protect against the potential benefit of simply increasing dose.*

# Supplemental 3: Disclosures unrelated to the published materials.

- Kathryn S Hayward: Supported by a National Health and Medical Research Council (NHMRC) of Australia Fellowship (#2016420) and Heart Foundation of Australia Future Leader Fellowship (#106607); Chief Investigator Medical Research Future Fund project grants (MRF2007425; MRF2024350; MRF2023177); Chief Investigator NHMRC project grant (1171890); Chief Investigator NIH project grant (R01NS115845); Chief Investigator NHMRC Centre of Research Excellence to Accelerate Stroke Trial Innovation and Translation (GNT2015705).
- Jessica Barth: The views expressed in this article are those of the authors and do not necessarily reflect the position or policy of the Department of Veterans Affairs or the United States government.
- Marian C Brady and the Nursing, Midwifery and Allied Health Professions Research Unit are funded by the Chief Scientist Office, part of the Scottish Government Health and Social Care Directorates, UK. MB is additionally supported by grant awards from the National Institute for Health Research grants (HSDR 132895 and 14/04/22; HTA 128829 and 10/135/02; RfPB 200739), Chest Heart and Stroke Scotland, the Stroke Association, UK and the Tavistock Trust for Aphasia, UK. The views expressed in this article are those of the authors and do not necessarily reflect the views of the funders.
- Leonid Churilov: Chief Investigator Medical Research Future Fund project grants; Chief Investigator NHMRC project grants; Chief Investigator on the NHMRC Centre of Research Excellence to Accelerate Stroke Trial Innovation and Translation.
- Jesse Dawson: Institutional research funding for trials of Vagus Nerve Stimulation (from MicroTransponder Inc), Robotic rehabilitation (from the UK NIHR and Chest Heart Stroke Scotland) and SaeboGlove therapy (Chest Heart Stroke Scotland and Chief Scientist Office Scotland). I have received conference registration from MicroTransponder Inc to present study data
- Sean Dukelow: Employed by the University of Calgary and Alberta Health Services. He has received operating grants from the Canadian Institutes of Health Research, Heart and Stroke Foundation of Canada and Brain Canada. He has also received speaking fees from Allergan and Merz, served on an advisory board for Ipsen and received consultancy fees from Prometheus Medical.
- Peter Feys. Consultancy for Roche. Supported by KBS, Promobilia, MS Society Flanders, FWO-MED.
- Catherine E. Lang: Supported by NIH R01HD068290, NIH R01MH723123, NIH R01NS101013, and NIH T32HD007434; Receives royalties from AOTA Press Inc.
