# Supplementary material for: Control intervention design for preclinical and clinical trials: Consensus-based core recommendations from the third Stroke Recovery and Rehabilitation Roundtable
Source: Int J Stroke. 2023 Oct 12;19(2):169–79. doi: 10.1177/17474930231199336 (PMC10811967; doi:10.1177/17474930231199336)
Supplement: sj-pdf-2-wso-10.1177_17474930231199336 – Supplemental material for Control intervention design for preclinical and clinical trials: Consensus-based core recommendations from the third Stroke Recovery and Rehabilitation Roundtable [file sj-pdf-2-wso-10.1177_17474930231199336.pdf]

3rd SRRR | Stroke Recovery and Rehabilitation Roundtable

Control intervention design for preclinical and clinical trials:

Consensus-based core recommendations from the third Stroke Recovery and Rehabilitation Roundtable (SRRR3).

DOI:10.1177/17474930231199336

https://redcap.link/SRRR-CONSIGN

RATIONALE

Control selection is a critical trial design issue: the benefit of an experimental intervention is established relative to a comparator. Often, little to no rationale is given for comparator choice, and fewer words and references are used to report a comparator vs. experimental intervention.

PURPOSE

To **understand the challenges for control design** and **produce a tool** to guide **control selection, description and reporting**.
If adopted, this tool could advance the science of stroke recovery and rehabilitation preclinical and clinical trials.

CONSIGN TOOL OVERVIEW

Step 1: Trial information
Research question, aim and hypothesis.

Step 2: Intervention reporting
Complete TIDieR for experimental intervention(s).

Step 3: Control design
Review and select appropriate control design(s).

Step 4: Common threats
Consider relevant threats and how to mitigate.

Step 5: Feasibility
Consider the feasibility aspects of selected control(s).

Step 6: Control reporting
Complete TIDieR for selected control(s).

RECOMMENDATIONS

Use the SRRR CONSIGN tool in trial design to support control comparator development (QR Code above).

Collaborate with biostatisticians, clinical, methodological experts and people with lived experience to optimise control comparator design.

Select an **optimal control comparator** that addresses the research question and statement of hypothesis, controls for the experimental intervention active ingredients, and mitigates possible threats to internal validity.

Describe the planned control comparator and the **process for monitoring** intervention adherence and fidelity **in the trial protocol** using TIDieR or an equivalent standard.

Describe the actual control comparator and the **extent to which it was delivered** as planned in a published trial report using TIDieR or an equivalent standard.

Document and report information specific to local, regional, and/or national systems of care to contextualise the control comparator, aid generalisation, and facilitate comparison to existing literature.
